# Differential growth of the northern Tibetan margin: evidence for oblique stepwise rise of the Tibetan Plateau

**DOI:** 10.1038/srep41164

**Published:** 2017-01-24

**Authors:** Fei Wang, Wenbei Shi, Weibin Zhang, Lin Wu, Liekun Yang, Yinzhi Wang, Rixiang Zhu

**Affiliations:** 1 State Key Laboratory of Lithospheric Evolution, Institute of Geology and Geophysics, Chinese Academy of Sciences, Beijing 100029, China; 2 CAS Center for Excellence in Tibetan Plateau Earth Sciences, Beijing 100029, China.

## Abstract

Models of how high elevations formed across Tibet predict: (a) the continuous thickening of a “viscous sheet”; (b) time-dependent, oblique stepwise growth; and (c) synchronous deformation across Tibet that accompanied collision. Our new observations may shed light on this issue. Here, we use ^40^Ar/^39^Ar and (U-Th)/He thermochronology from massifs in the hanging walls of thrust structures along the Kunlun Belt, the first-order orogenic range at the northern Tibetan margin, to elucidate the exhumation history. The results show that these massifs, and hence the plateau margin, were subject to slow, steady exhumation during the Early Cenozoic, followed by a pulse of accelerated exhumation during 40–35 Ma. The exhumation rate increases westward (from ~0.22 to 0.34 and 0.5 mm/yr). The two-fold increase in exhumation in the western part (0.5 mm/yr) compared to the eastern part suggests westward increases in exhumation and compressional stress along the Kunlun Belt. We relate these observations to the mechanisms responsible for the oblique stepwise rise of Tibet. After collision, oblique subduction beneath Kunlun caused stronger compressional deformation in the western part than in the eastern part, resulting in differential growth and lateral extrusion.

Although various models or hypotheses have been developed to describe the manner of Tibetan Plateau growth^1^,^2^,^3^,^4^,^5^, the link between plateau growth and the geodynamics of intracontinental deformation remains at the center of debates. The ongoing convergence between India and Eurasia has produced double-thickness crust with thicknesses averaging ~60 km over a distance ~2000 km north of the plate boundary. The viscous sheet model^3^ and the plastic, or oblique step-wise growth, model^1^,^2^ are based on different rheologies (continua vs. rigid blocks separated by faults) but predict a scenario of northward propagation of strain and younging ages. However, these hypotheses have been challenged recently. New studies show that the growth of the Tibetan Plateau may have begun in central Tibet and expanded to encompass most of Tibet, which is the central Tibet growth model^4^,^6^,^7^,^8^. Although the onset time of growth is still elusive, most studies suggest that the thickened crust and high topography in central Tibet and its northern margin were built up by 40–50 Ma^4^,^5^,^6^,^7^,^8^,^9^,^10^,^11^,^12^ (Fig. 1), which is close to the commonly suggested collision time of ~47–55 Ma^2^,^13^,^14^,^15^. These data appear to contradict the notion of initiation and propagation of strain away from the collision boundary. However, recent re-dating of the initial collision suggests that the collision time should be moved to ~65–60 Ma ago^16^,^17^,^18^. This proposed collision time is ~30–25 million years earlier than the onset of regional deformation in central Tibet and the northern margin, instead of being synchronous with it.

Cenozoic terrains along the northern margin of the Tibetan Plateau are considered to be the consequence of the far-field effects of continued Eurasia–India convergence^2^ and can be regarded as ideal locations to test the growth models. Characterized by high elevation accompanied by low relief in the western part and low elevation accompanied by high relief in the eastern part^19^, the Kunlun Belt is the first mountain belt defining the northern Tibetan margin. Composed mainly of Devonian to Early Triassic marine sediments, Jurassic and Cenozoic non-marine rocks^20^,^21^,^22^ and pre-Cenozoic granitoids (Fig. 2), the Kunlun Belt is considered, from a tectonic perspective, to be a part of the Paleozoic-Triassic collision belt and was rejuvenated during the Cenozoic Eurasia–Indian collision and the growth of Tibetan Plateau^11^,^23^. A few low-temperature thermochronological studies have been reported from the eastern part of the Kunlun Belt^5^,^21^,^24^,^25^,^26^, where a cooling history between 350–60 °C was extracted. A rapid exhumation event and therefore the onset of deformation during the Cenozoic were then concluded to have occurred at approximately 30 Ma^24^,^25^ or 40 Ma^5^,^10^ in the eastern part of the Kunlun Belt. In contrast, low temperature geochronological studies are lacking, and existing constraints on the exhumation and cooling history of the western part of the Kunlun Belt are very poor. Therefore, although it is the first-order orogenic belt of the northern Tibetan margin and has great potential to reveal the evolution of the plateau, systematic research and direct evidence of the couplings between tectonics and surface processes are still lacking, and the modes of uplift and exhumation needs to be better quantitatively constrained.

To evaluate the timing and history of mountain-building in the Kunlun Belt, we date and model samples from three new vertical age-elevation transects from different parts of the Kunlun Belt (transect 1 comes from the middle part; transects 2 and 3 are found in the western part) (Fig. 2, Table S1). To facilitate comparisons with the eastern part of the Kunlun Belt, the original data from two of our previous transects in the eastern part (transects 4 and 5, Fig. 1)^10^ are also processed (Table S1) and modeled in this study. All of these transects are distributed evenly over a distance of ~600 km along the Kunlun Belt and are located in the immediate hanging wall of the North Kunlun Fault (Fig. 1). Thus, the exhumation history of the range provides an important constraint on the manner of mountain-building along the northern Tibetan margin and can potentially inform models of the growth of the plateau.

## Results

We present results using the K-feldspar ^40^Ar/^39^Ar and apatite (U-Th)/He thermochronological systems in Figs 3 and 4 and Supplementary Tables S3 and S4, respectively. A summary of the ages, including their sample locations, is provided in Table S1. Furthermore, the kinetics of argon and helium diffusion in these systems span a temperature window ranging from ~350 °C down to ~60 °C^27^,^28^ and allow us to investigate the thermal history of this part of the plateau margin in unprecedented detail.

### K-feldspar ^40^Ar/^39^Ar analysis results

The results of K-feldspar ^40^Ar/^39^Ar analysis of two samples from transects 1 (15kl03) and 2 (1335-2), respectively, are shown in Fig. 3 and Supplementary Table S3. Two K-feldspar samples exhibit flat age spectra (Fig. 3), defining good plateaus that account for >80% of the total ^39^Ar released at high temperature steps, implying that rapid cooling occurred at these times (see Supplementary Information for details). The plateau ages are 232.5 ± 1.4 Ma (15kl03) and 234.6 ± 1.2 Ma (1335-2), which are similar to those reported by previous studies in the Kunlun Belt^11^,^14^,^15^. These massifs were rapidly exhumed during the early Late Triassic and are attributed to the orogeny of the Kunlun Belt, which was probably driven by the collision between the Songpan-Ganzi and Kunlun blocks^11^,^29^.

Conversely, the two age spectra display staircase shapes at the low temperature steps of the step-heating (Fig. 3). Although less than 20% of the total released ^39^Ar resides within these low-temperature domains (Fig. 3), the behavior^30^,^31^,^32^,^33^ of multi-diffusion domains within K-feldspar suggests that these staircase spectra are the result of slow cooling and, therefore, record cooling ages between ~350–150 °C. Their minimum ages of 198.8 ± 2.4 and 90.3 ± 2.1 Ma (Fig. 3, Table S1) reflect the final time when the samples passed the ~150 °C isotherm.

The ^40^Ar/^39^Ar thermochronologic data from K-feldspars imply that rocks now at the surface resided at or above temperature of ~350 °C during the early Late Triassic and ~150 °C during the late Cretaceous. These results place a maximum bound on total exhumation of ~11 km since the middle Triassic and ~5 km since the late Cretaceous, assuming a gradient of 30 °C/km^5^.

### Apatite (U-Th)/He dating results

Ages from the apatite (U-Th)/He thermochronological system provide insight into the Cenozoic cooling history. Single apatite grains are analyzed by using the (U-Th)/He dating technique. The results are presented in Fig. 4 and Supplementary Table S4.

(U-Th)/He ages from transect 1 range from 47.4 ± 4.5 Ma at 4301 m down to 29.5 ± 2.8 Ma at 3125 m (Table S1, Fig. 4a), exhibiting a distinct age-elevation trend. The pattern of the trend shows an increase in the gradient at approximately 35 Ma, implying that accelerated exhumation began at this time. This observation is similar to those from our previous transects in the eastern part of the Kunlun Belt near Nuomuhong^10^, where a rapid exhumation starting around 35 Ma was also recognized. In contrast, (U-Th)/He ages from transect 2 present a steep age-elevation relationship: the ages ranging from 39.8 ± 3.0 to 36.6 ± 3.2 Ma are dispersed over an elevation range from 4727 m down to 4102 m (Fig. 4b), with an average age of 38.3 ± 3.2 Ma. Similarly, three samples with almost the same age (43.1 ± 4.5 Ma, 43.2 ± 4.5 Ma and 43.4 ± 4.4 Ma) from transect 3 are distributed over a vertical range of 4373–3892 m (Fig. 4c). The relatively small elevation ranges (~500–600 m) from transect 2 and 3, which are partially responsible for the age clustering, are insufficient to permit examination of age-elevation correlations for these massifs. Even so, given that the seven samples do not show any trend reflective of a slow exhumation in the age-elevation correlation suggest that transects 2 and 3 reflect parts of accelerated exhumation trend, although we do not see the break where it starts. This can be demonstrated using our new (U-Th)/He dates from detrital apatites (39.5–43.2 Ma, authors’ paper under preparation) from Miocene growth strata in the Kumkuli intramontane basin, which suggest that the apatites eroded from the surrounding mountains (the western part of the Kunlun Belt and Qimantagh) have the same ages as transects 1 and 2. In addition, transect 2 exhibits very slightly negative correlation between age and depth (Fig. 5, Table S1), implying that this massif could have been tectonically tilted slightly. However, such an event is difficult to interpret on the basis of just this marginal correlation. Therefore, we conclude that the patterns of transects 2 and 3 most likely represent a monotonic cooling or an accelerated exhumation between ~38 and ~43 Ma.

**Figure 1:**
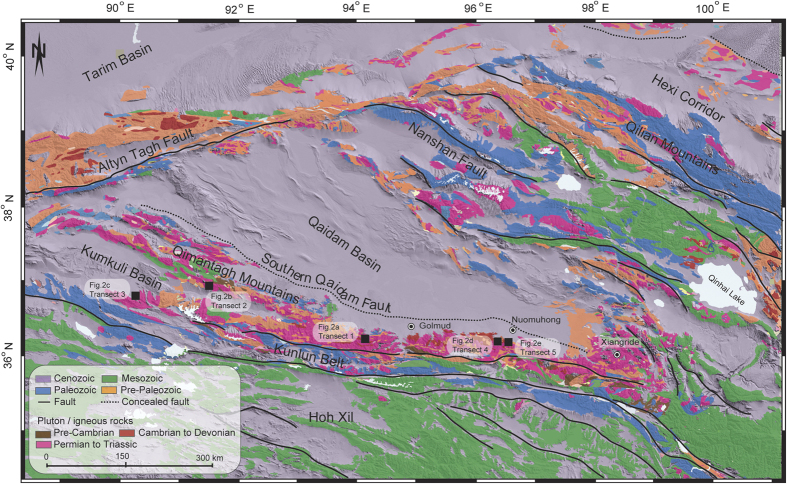
Simplified regional geologic map of the central and northern margin of the Tibetan Plateau and its adjacent regions. Tectonostratigraphic terranes are shown in the explanation. Figure was generated using MapInfo Professional [11.0.4], (URL: http://www.pbinsight.com/) and Global Mapper [17.2], (USL: http://www.bluemarblegeo.com). The coordinate system is the World Geodetic System 1984 (WGS84). To facilitate comparisons with the eastern part of the Kunlun Belt, two transects from our previous work^10^ are shown as transect 4 and 5.

**Figure 3:**
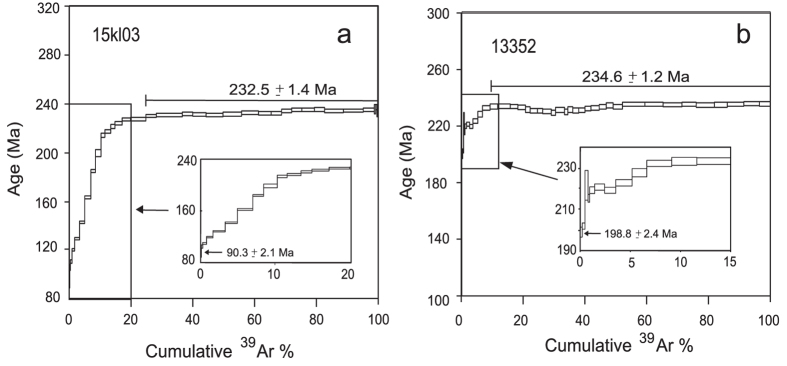
^40^Ar/^39^Ar results of k-feldspars. Plateau ages are shown at high temperatures. Insets indicate the staircase age spectra of K-feldspar at low temperatures with minimum ages labeled.

**Figure 4:**
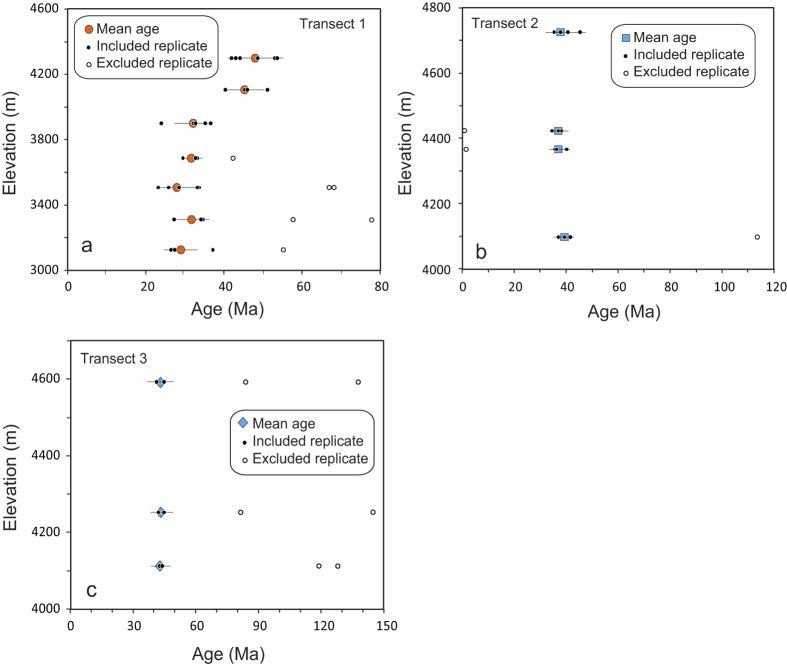
Age-elevation transects for (U-Th)/He thermochronologic data. Error bars denote 2σ analytical uncertainties. (**a**), (U-Th)/He ages (circles) for transect 1; (**b**), (U-Th)/He ages (squares) for transect 2; (**c**), (U-Th)/He ages (diamonds) for transect 3. Filled symbols denote grain replicates included in the mean age determination. Open symbols denote excluded grain ages.

**Figure 5:**
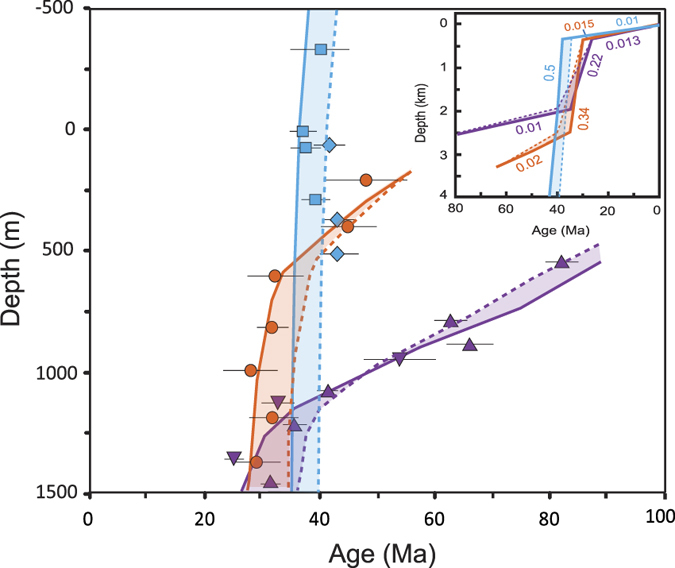
Composite age-depth transects comparing data with predictions of forward thermal models. Uncertainties are shown as 2σ. Inset shows color-matched time-depth histories of transects used in models. Numbers denote average exhumation rates in mm/yr for each stage. Exhumation histories that represent acceleration starting 35 Ma (solid lines) or 40 Ma (dashed lines) are calculated. Shaded region between two histories represents of solutions between these time periods. Transects 4 (upper triangles) and 5 (lower triangles) from our previous work^10^ were modeled as well to make comparison. Time-depth history solution with acceleration starting 35 Ma fits transect 1, 4 and 5 perfectly, while the solution with acceleration starting 40 Ma fits transect 2 and 3 well.

These results clearly reveal a picture of differential evolution of exhumation history along the Kunlun Belt. To compare directly, samples from each transect are normalized to the local elevation of a remnant erosion surface resolved from a 30-m resolution Digital Elevation Model (DEM) (Figs S1 and S2, see Supplementary Information), and plotted as age versus structural depth (Fig. S3, Fig. 5). To permit comparisons between different parts of the Kunlun Belt, two of our previous transects near Nuomuhong^10^, which are labeled as transects 4 and 5 in Fig. 1, are normalized to the local erosion surface (Table S1) and plotted and modeled in Fig. 5 as well. The five transects form three distinct age-depth trends in the age-depth plot (Fig. 5). Given various scenarios, forward thermal modeling was carried out on each trend, and the solutions are shown in Fig. 5 and Fig. S6. The data and modeling results demonstrate a tendency for uplift and exhumation to strengthen from east to west along the Kunlun Belt during Eocene time. The gentle slopes of transects 1, 4 and 5 imply that a very slow, constant exhumation (0.01–0.02 mm/yr) history was predominant in the Kunlun Belt during the late Cretaceous to early Paleogene (Fig. 5). Abrupt changes in the slopes of transects 1 (from 0.02 to 0.34 mm/yr), 4 and 5 (from 0.01 to 0.22 mm/yr) appear at 35 Ma, indicating that the onset of accelerated exhumation occurred 35 Ma ago in the eastern and middle parts of the Kunlun Belt. Similarly, modeled results that form a steep slope at ~40 Ma in transects 2 and 3 demonstrate that rapid exhumation occurred at this time at a rate of 0.5 mm/yr (Fig. 5). Although the difference (~5 Myr) in starting times of accelerated exhumation between the eastern and western parts of the Kunlun Belt is small and can be obscured by the uncertainties of the dates, the overall trend illustrated by all samples from transects 2 and 3 (where no samples yielded ages <35 Ma) suggests that this difference may be meaningful and reflects the “true” situation. More evidences are discussed later that show that this inference is reasonable. The data from transects 2 and 3 do not record the abrupt change in slope, most likely because of the rapid exhumation which removed overlying rocks that would have recorded rapid slope changes in those transects. Nevertheless, the uppermost sample of this steep slope indicates the lower limit of rapid slope change in depth, which is 900 m and 1400 m higher than those of transect 1 and transects 4 and 5, respectively (Fig. 5). According to He kinetic and diffusion theory, the rapid slope change in the helium age-depth curve defines the start of the rapid motion of rocks towards the Earth’s surface when pass the closure isotherm (~60 °C)^28^. Therefore, our observations suggest that rapid uplift and exhumation occurred earlier in the western part than in the eastern part of the Kunlun Belt during the early Cenozoic.

## Discussions

Our new data suggest that the Kunlun Belt grew differentially from west to east during the Cenozoic. Specifically, uplift initiated at approximately 40 Ma in the western part and at approximately 35 Ma in the eastern part. Exhumation rates of 0.22 mm/yr in the eastern part, 0.34 mm/yr in the middle part and 0.5 mm/yr in the western part suggest a westward increase in uplift, under the logical assumption that the different parts of the Kunlun Belt experienced the same climatic conditions. Given a uniform geologic setting, the westward strengthening of uplift and exhumation suggest that lateral compression increased in strength from east to west along the Kunlun Belt. The modeled exhumation rates (Fig. 5) imply that the western part experienced compressive forces twice as strong as those experienced by the eastern part.

A growing body of evidence indicates that the regional deformation was stronger and initiated earlier in the western part than in the eastern part along the Kunlun Belt during early Cenozoic. Rapid exhumation of the eastern part of the Kunlun Belt at approximately 30 Ma is inferred from accelerated cooling determined from feldspar multi-domain diffusion modeling^24^,^25^. Previous (U-Th)/He data revealed a rapid exhumation event beginning at approximately 35 Ma in the eastern part of the Kunlun Belt^5^. Apatite and zircon (U-Th)/He and apatite fission-track ages along the strike of the eastern part of the Kunlun Fault show that exhumation rates increase eastward, from 30–25 Ma near Golmud to 20–15 Ma near Xiangride^34^ (Fig. 1).

The initiation of sedimentation in the Kumkuli basin since the middle Eocene suggests that rapid exhumation started ~40 Ma in the western part of the Kunlun Belt^35^. Detrital apatite (U-Th)/He ages that cover a range of 39.5–43.2 Ma (authors’ paper under preparation) from Miocene growth strata in the Kumkuli Basin also indicate that rapid exhumation started at approximately 40 Ma in the western part. Sedimentation rates in the adjacent Qaidam Basin increased rapidly during the period between 40–36.6 Ma^25^,^36^,^37^,^38^. Growth strata that were deposited approximately 40 Ma are extensively preserved in the peripheral areas of the western Qaidam Basin^35^,^39^,^40^ implying rapid denudation of the surrounding mountains. A detailed study of the magnetostratigraphy of the lower Tertiary sedimentary sequence in the western Qaidam Basin shows that the sedimentation initiated sometimes before 40 Ma^41^. The main depocenter of the Qaidam Basin shifted from the western part to the eastern part during the late Cenozoic^35^, suggesting that the provenance of sedimentation moved from west to east in the surrounding mountains. Regional seismic reflection profiles across the Qaidam Basin reveal that a progressive shift in crustal thickening mechanisms: crustal shortening decreases from >48% in the west to <1% in the east during the early Cenozoic^42^. In the Hoh Xil basin, immediately south of the study area, there was rapid sedimentation at approximately 40–35 Ma^4^. A recent review that investigated various lines of evidence, including deformation, faulting, the results of basin research, paleomagnetic declination anomalies and thermochronology, concluded that growth and deformation of the northern Tibetan margin began at 50–40 Ma^11^,^12^.

Two end-member models of how the Tibetan Plateau formed are: (a) diffuse crustal thickening of a viscous sheet, with shear on vertical planes playing a subsidiary role (i.e., the viscous sheet model)^3^, and (b) localized shear between rigid lithospheric blocks (i.e., oblique stepwise growth model)^1^. Both models predict time-dependent, northward progression of strain and deformation that is initially concentrated at the plate boundary and then propagates in time away from that boundary as topographic stresses begin to favor deformation at greater distances from the plate boundary.

Recently, a new mechanism for Tibetan Plateau growth has been proposed, namely the central Tibet growth model, although when and how are still in dispute. The main feature of this model is that the region of high topography initiated in central Tibet and the lithospheric stresses propagated north and south from this high topography outward^4^,^6^,^7^,^8^,^11^. Although the onset time of growth remains controversial, most studies suggest a scenario in which the high topography in central Tibet and the northern Tibetan margin was built up by 40–50 Ma^4^,^6^,^7^,^8^,^11^,^12^.

The timing of collision between India and Eurasia has been disputed over the past several decades but is crucial for understanding the far-field effects of the collision. The commonly suggested age of collision of 47–55 Ma^2^,^13^,^15^ implies an immediate response to the collision in central and northern Tibet. However, several recent studies suggest that the collision between India and Eurasia may have occurred during the early Paleocene (~65–60 Ma)^16^,^17^,^18^, 30–20 Myr earlier than the initiation of regional scale deformation across the central and northern Tibet. These latest observations imply that the propagation of the far-field effects of the collision to the northern margin was much slower than previously thought^2^,^5^. (U-Th)/He ages from the North Qilian Shan suggest that rapid exhumation extended slowly northward, reaching as far as the Hexi Corridor by the late Miocene (~10 Ma^43^), and apatite fission track ages from the Liupan Shan indicate that rapid exhumation did not begin until ~8 Ma^44^. Although a complete picture of how the deformation crossed the Tibetan Plateau remains elusive, diachronous initial growth in different portions of the plateau (40 Ma ago in the east and 20 Ma ago in the west^6^; 40 Ma ago in the north^4^; 30 Ma ago in the southeast^45^) imply that strain propagation may have had a key role in the evolution of Tibet.

The viscous sheet model predicts progressive northward migration of the associated crustal thickening and therefore synchronous uplift and uniform force at the frontline in the propagation direction after the rigid “indenter” represented by India converged with the weaker Eurasian lithosphere. However, this is not consistent with our observations of diachronous deformation with different intensities across the northern Tibetan margin. In addition, the strain generated by edge tractions in the thin viscous sheet model is insufficient to generate faulting in northern Tibet^5^.

Our thermochronologic data and modeling results from the Kunlun Belt support the oblique step-wise growth mechanism. Although how and when the stress propagated across the Tibet is called into question by new deformation evidence from central Tibet^4^,^6^,^7^,^8^,^12^, the notion of the oblique stepwise growth model^2^, i.e., the oblique rise of Tibet and lateral extrusion of the thickened crust, is consistent with our thermochronologic results along the Kunlun Belt. This model describes a picture of oblique subduction after collision. Oblique subduction of the Asian lithospheric mantle plays a key role that involves both lateral extrusion and crustal thickening^2^. It can account for the markedly asymmetric growth of relief toward the east and the eastward extrusion observed in northeastern and eastern Tibet^45^. Far-field impacts on the northern Tibetan margin initially developed approximately 40–35 Ma. Oblique subduction of the Qaidam block beneath the Kunlun Belt to depth of approximately 200 to 300 km^2^ resulted in sinistral slip along the Kunlun and North Kunlun Faults and crustal thickening. A stronger stress was created in the western than in the eastern part of the Kunlun Belt due to the northeast-southwest oblique compression, resulting in an eastward decrease in uplift and exhumation along the Kunlun Belt (Fig. 5). The movement along lithospheric faults resulted in eastward extrusion of the Tibetan Plateau, followed by a major compression event in the late Eocene. Consequently, the present-day Kunlun Belt contains a highland with low relief in the western part and a lowland with high relief in the eastern part.

Our results support the temporal development predicted by the central Tibetan growth model. This model suggests a scenario of plateau growth in central Tibet and propagation to the northern Tibetan margin by 40–50 Ma that is consistent with our observations along the Kunlun Belt^4^,^6^,^7^,^8^,^12^. The growth of central Tibet and northward stress propagation are associated with folding and thrusting that resulted from subduction of the Indian lithosphere underneath the Tibetan plateau^4^,^6^,^7^,^8^. Northward thrusting from central Tibet is obliquely oriented^2^,^4^,^6^,^8^, causing an eastward decrease in compressional stress along the Kunlun Belt.

## Methods

### ^40^Ar/^39^Ar geochronology

Characterized by complicated microstructures that serves as domains with different sizes, argon retention properties and closure temperatures^30^,^31^,^46^, K-feldspars are potentially able to record ^40^Ar/^39^Ar ages of closure temperatures ranging from 350 down to 150 °C as they cool^27^,^47^, and thermal histories can consequently be extracted^30^,^31^,^32^,^33^,^48^. A high resolution (36–40 steps) step-heating technique was applied from 450 to 1300 °C in a ^40^Ar/^39^Ar analysis to reveal the argon distributions within the K-feldspar grains in as much detail as possible (Table S3). K-feldspars were carefully inspected to meet the criteria and requirements of thermochronology using the ^40^Ar/^39^Ar method^27^,^48^. The sample processing and laboratory procedures involved in ^40^Ar/^39^Ar analysis are described by Wang *et al*.^31^. Ages were calculated against the international standard YBCs sanidine (29.286 ± 0.045 Ma^49^). The experiments were conducted on an MM5400 mass spectrometer; plateau ages were calculated using adjacent ages of step-heating that agreed within a range of 2σ. A complete discussion of sample preparation and analytical procedures can be found in the Supplementary Information.

### (U-Th)/He geochronology

See the Supplementary Information for sample preparation. In principle, clear, euhedral apatite grains longer than 130 μm and wider than 75 μm were used for (U-Th)/He dating. Each apatite grain was inspected carefully under a high-power microscope to eliminate those containing impurities and inclusions. Needle-like, fragmentary, subhedral, rounded or zoned apatite grains were excluded as well. After this selection process, each selected apatite grain was enclosed in a 1 mm × 1 mm platinum capsule and moved one after another into a well in a stainless-steel disk for He measurement using an Alphachron MK II noble gas mass spectrometer. Each grain was heated twice at 900 °C by a diode laser, each time for 10 min, to extract the He completely as possible. The abundance of ^4^He was determined using the isotope dilution technique. The ^3^He spike used is calibrated daily against an independent ^4^He standard tank. The uncertainty of the ^4^He measurements averaged less than 2%. After completion of the He measurements, the degassed apatite grains were removed and prepared for U and Th measurements. After being removed from the capsule and placed in a beaker, each apatite was dissolved in 25 μL of a liquid reagent made up of 50% HNO_3_ spiked with ^235^U and ^230^Th. The beakers were subjected to ultrasound vibrations until the apatite crystals had dissolved completely. Finally, the liquid reagent was diluted to 5% HNO_3_ and analyzed for U and Th abundances by using a Thermo Fisher X-Series II ICP-MS. As determined by averaging replicate analyses of spiked standard solutions, the analytical precisions of the measured ^235^U/^238^U and ^230^Th/^232^Th ratios were 0.8% and 0.5%, respectively. A series of Durango standard grains was analyzed between measurements of sample grains for monitoring the whole experimental protocol. Application of (U-Th)/He methods at IGGCAS yielded an averaged Durango apatite age of 32.24 ± 1.01 Ma^50^ with an internal precision (1σ) of 1.5%. This age is quite consistent with the recommended age^10^,^50^. A detailed description of analytical procedures can be found in the Supplementary Information.

### Forward modeling

A one-dimensional thermal model was utilized for forward modeling of the thermal response to exhumation. Various exhumation histories were imposed, subject to the constraint imposed by a thermal gradient of 30 °C/km (see the Supplementary Information), and subject to a constant surface temperature of 10 °C. Thermal history solutions for each transect were obtained using a constant temperature offset (30 °C/km) model in QTQt v.5.3.0^51^, and the median thermal history was used for the transect. The detailed modeling procedure can be found in Gallagher *et al*.^51^. Samples along a transect are used to calculate model helium ages using the helium diffusion kinetics from Flowers *et al*.^52^ (radiation damage model RDAMM). We seek a series of preferred model fits that faithfully represent most of the sample ages, i.e., the basic trend of the data. A detailed description of the modeling procedure can be found in the Supplementary Information.

## Additional Information

**How to cite this article**: Wang, F. *et al*. Differential growth of the northern Tibetan margin: evidence for oblique stepwise rise of the Tibetan Plateau. *Sci. Rep.* **7**, 41164; doi: 10.1038/srep41164 (2017).

**Publisher's note:** Springer Nature remains neutral with regard to jurisdictional claims in published maps and institutional affiliations.

## Supplementary Material

Supplementary Information

## Figures and Tables

**Figure 2:**
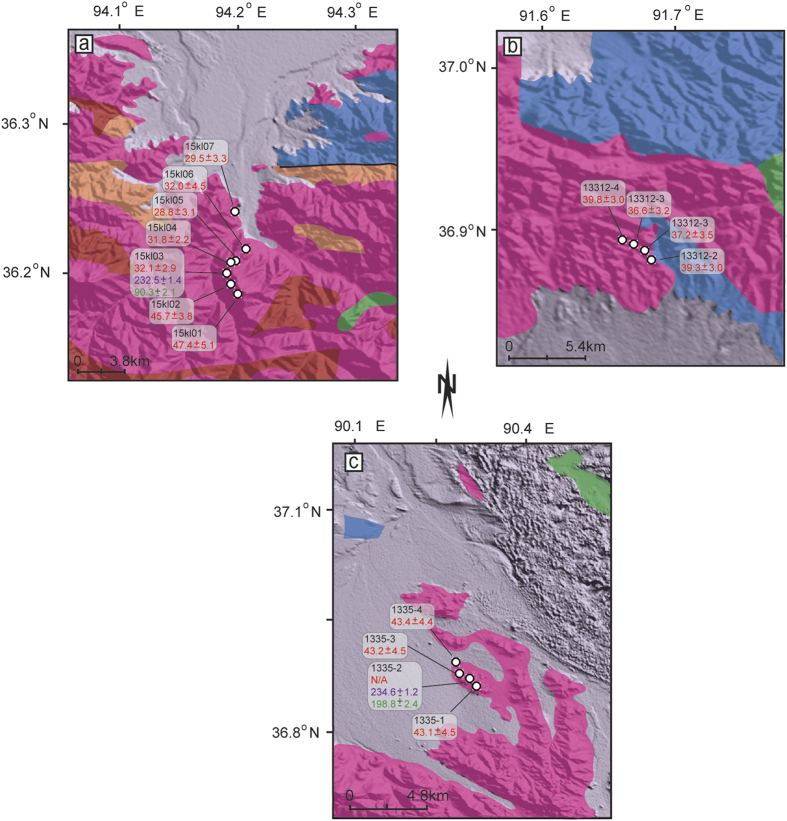
Geologic map of the transects of massifs sampled. Granitic massifs in the hanging wall of North Kunlun Fault zone. Figures were generated using MapInfo Professional [11.0.4], (URL: http://www.pbinsight.com/) and Global Mapper [17.2], (USL: http://www.bluemarblegeo.com). The coordinate system is the World Geodetic System 1984 (WGS84). (**a**) The massif of transect 1 60 km to the west of Golmud; (**b**) Massif of transect 2 in the west part of Kunlun belt (Qimantagh); (**c**), Massif of transect 3 in the west part of Kunlun belt. Explanation of geologic units as in Fig. 1. Numbers in red are the (U-th)/He age in Ma, while numbers in purple and green are plateau and minimum ages of ^40^Ar/^39^Ar in Ma, respectively.
