# Supplementary material for: Differential growth of the northern Tibetan margin: evidence for oblique stepwise rise of the Tibetan Plateau
Source: Sci Rep. 2017 Jan 24;7:41164. doi: 10.1038/srep41164 (PMC5259709; doi:10.1038/srep41164)
Supplement: Supplementary Information [file srep41164-s1.doc]

Supplementary Information for

Differential growth of the northern Tibetan margin: evidence for oblique stepwise rise of the Tibetan Plateau

Fei Wang^a,b^,*, Wenbei Shi^a^, Weibin Zhang^a^, Lin Wu^a^, Liekun Yang^a^, Yinzhi Wang^a^, Rixiang Zhu^a^

^a^ State Key Laboratory of Lithospheric Evolution, Institute of Geology and Geophysics, Chinese Academy of Sciences, Beijing 100029, China

^b^ CAS Center for Excellence in Tibetan Plateau Earth Sciences

* Corresponding author: Fei Wang (wangfei@mail.iggcas.ac.cn)

**Geological setting and sample collection**

Extending over 1,000 km from east to west, the Kunlun Belt bounds the Tibetan Plateau in the south and the Qaidam Basin in the north (Fig.S1). Composed mainly of Devonian to Early Triassic marine sediments, Jurassic and Cenozoic non-marine rocks and pre-Cenozoic granitoids (Fig.S1), the Kunlun Belt is considered, from a tectonic perspective, to be a part of the Paleozoic-Triassic collision belt and was rejuvenated during the Cenozoic Eurasia–Indian collision and the uplift of the Tibetan Plateau^1-4^. The granitoid rocks are dated as Early Cambrian to Early Devonian (515 - 393 Ma ^2, 3^) and Late Permian to Triassic (261–215 Ma ^3,5-7^). As a result of northwards subduction of the Tethys oceanic plate under the Kunlun Terrane and the consequent collision between the Songpan-Ganzi and Kunlun blocks ^7,8^, the Kunlun Belt was built up during the Late Permian to Middle Triassic.

Although various models or hypotheses have been developed to describe the manner of Tibetan Plateau growth ^9-13^, the link between plateau growth and the geodynamics of intracontinental deformation remains at the center of debates. The ongoing convergence between India and Eurasia has produced double-thickness crust with thicknesses averaging ~60 km over a distance ~2000 km north of the plate boundary. The viscous ^11^ and plastic models ^9,10^ are based on different rheologies (continua vs. rigid blocks separated by faults), but predict a scenario of northward propagation of strain and younging ages. However, these hypotheses have been challenged recently. Isotopic data from central Tibet and its northern margin suggest that the thickened crust and high topography were built early and synchronously (~40 -50 Ma) in the collision history ^12-15^, which is similar to the collision time of ~47-50 Ma ^16^. These data appear to contradict the notion of initiation and propagation of strain away from the collision boundary. Recent re-dating of the initial collision suggests that the collision time should be moved to ~65-60 Ma ago ^17-19^. This proposed collision time is ~30-25 million years earlier than the onset of regional deformation in central Tibet and northern margin, instead of being synchronous with it ^13^. These new results imply that the northward strain propagation across the Tibetan Plateau from the convergence boundary after collision is viable.

To evaluate the timing and history of mountain-building in the Kunlun Belt, we date and model samples from three new vertical age-elevation transects from different parts of the Kunlun Belt (transect 1 comes from the middle part; transects 2 and 3 are found in the western part) (Fig. 2, Table S1). To facilitate comparisons with the eastern part of the Kunlun Belt, the original data from two of our previous transects in the eastern part (transects 4 and 5, Fig. 2) ^15^ are also modeled in this study. All these transects are distributed evenly over a distance of ~600 km along the Kunlun Belt and are located in the immediate hanging wall of the North Kunlun Fault (Fig. 2). Thus, the exhumation history of the range provides an important constraint on the manner of mountain-building along the northern Tibetan margin and can potentially inform models of the growth of the plateau.

Samples were collected from extensive outcrops of Permo-Triassic granitic rocks, and thus we were able to collect vertical transects on with minimal horizontal offset where possible. Seven samples were collected from transect 1 from a massif 60 km to the west of Golmud; four samples were collected from transect 2 from the Qimantagh Mountains and four samples were collected from transect 3 in the western part of the Kunlun Belt. As a branch of the west part of the Kunlun Belt, the Qimantagh Mountains are inferred to have been built up during the reactivation of the Kunlun belt induced by Tibetan rising ^4^. Besides, the Qimantagh Mountains is located in the hanging wall of the North Kunlun fault as well. Our transect 2, although from the Qimantagh Mountains, is very close to the west part of the Kunlun Belt (Fig.1), and hence close to transect 3. More importantly, our data show that synchronous rapid exhumation occurred in the Qimantagh Mountains and the west part of the Kunlun Belt, suggesting that they have experienced the same evolution history during the Cenozoic. In the eastern part of the Kunlun Belt, two of our previous transects ^15^ located south of Nuomuhong, here labeled as transects 4 and 5 (Fig.S1), were used for comparison.

**From elevation to depth**

The transects studied are located within a range of ~ 600 km (Fig. S1), and they are very likely impacted by the effects of long-wavelength topography and different amounts of throw along the North Kunlun Fault. The effect of long-wavelength undulations in the geomorphic surface is a major factor that controls the undulation of isotherms beneath the surface ^20^. Therefore, the differences in depth of the same isotherm caused by long-wavelength undulations between different locations should be corrected for when direct comparisons of elevation-age transects from different locations are made ^13^.

A regional erosion surface preserved throughout the Kunlun Mountains was recognized ^13,21^ from DEM data and used as the geomorphic surface in the correction of thermochronological transects from different locations ^13^. The erosion surface could have been formed as early as the Eocene because it cut part of Eocene sedimentary strata ^21^, and likely represents a horizontal datum prior to accelerated exhumation event along the Kunlun belt at 40 - 45 Ma ^13^. A lifted erosion surface is regarded as a mark of terrain uplift ^22^ because it originally forms near the regional base level of erosion. When uplift and consequently erosion occur, the surface is elevated and then dissected by erosion, leaving surface remnants in the highest reaches of mountains.

By using a 30-m resolution DEM data, we identified remnants of this erosion surface, which do not show local tilting, near the studied transects (Fig. S2). The altitudes of these surfaces vary by ±220 m, i.e., they are 4550 m near transect 1 (Fig.S2a, b), 4430 m near transects 2 (Fig.S2c), 4650 m near transect 3(Fig.S2d), and 4600 m near transects 4 and 5 (Fig.S2e,f). The perpendicular distance from the sample elevation to the surface was measured, and data from each transect was plotted as age versus structural depth to the local elevation of this geomorphic surface (Fig. S3).

**Sample preparation**

The granitic rock samples were crushed and sieved to obtain coarser fragments between 280 and 450 μm and finer fragments between 200 and 75 μm in diameter. K-feldspar grains free of impurities and inclusions were hand-picked under a microscope from the coarser fragments for ^40^Ar/^39^Ar analysis, whereas the apatite crystals were manually selected from the finer fragments and carefully inspected under a high-power microscope for (U-Th)/He dating. The selected grains were rinsed three times ultrasonically in deionized water, rinsed in acetone, and then dried for analysis. All experiments were conducted at the ^40^Ar/^39^Ar and (U-Th)/He Laboratory of the Institute of Geology and Geophysics at the Chinese Academy of Sciences (IGGCAS).

**^40^Ar/^39^Ar geochronology method**

All aliquots of k-feldspar grains were wrapped in aluminum foil to form wafers, and stacked in quartz vials together with the international standard YBCs sanidine (29.286 ± 0.045 Ma) ^23^. Neutron irradiation was carried out in the 49-2 Nuclear Reactor (49-2 NR), Beijing (China), with a neutron flux per hour (J/h) of 0.00021 ^24^. Interfering nucleogenic reactions were checked for every irradiation by using CaF and K_2_SO_4_, and the correction factors in this study were [^36^Ar/^37^Ar]_Ca_=0.000261±0.000014; [^39^Ar/^37^Ar]_Ca_= 0.000724±0.000028; and [^40^Ar/^39^Ar]_K_=0.000880±0.000023.

Sample wafers were then placed into a Ta tube resting in the Ta crucible of an automated double-vacuum resistance furnace. High resolution ^40^Ar/^39^Ar measurements were performed by using mass-spectrometer MM5400 at the ^40^Ar/^39^Ar Laboratory of the Institute of Geology and Geophysics of Chinese Academy of Sciences (IGGCAS), Beijing. Mass discrimination was monitored using an on-line air pipette from which multiple measurements were made before and after each incremental-heating experiment. The mean over this period was 1.00831±0.00017 per amu and the uncertainty of this value was propagated into all age calculations. The experiments were scheduled on a high resolution step-heating scheme, starting from 450 ^o^C until 1300 ^o^C. Hot system blanks determined several times each day prior to degassing the samples were typically 3×10^-16^ mols of ^40^Ar and 9×10^-19^ mols of ^36^Ar, 2-3 orders of magnitude smaller than the sample signals.

Plateau age was determined from five or more contiguous steps with concordant ages at 95% confidence level, comprising >80% of the total ^39^Ar released. The data were processed using ArArCALC ^25^, and the external uncertainties arising from the procedure were propagated into the final result. Errors here are reported at the 2σ confidence level.

**(U-Th)/He geochronology method**

For the helium analyses, we used a fully automatic He extraction system called the Alphachron MK II, which is produced by Australian Scientific Instruments Pty, Ltd. (ASI). The system consists of a 970 nm diode laser, a fully automatic gas purification line, and a quadruple mass spectrometer. Multiple analyses suggest that 10 A (heating to a temperature of 850–900 °C) laser heating for 5 min was sufficient to extract 99% of ^4^He from the heated crystals. Replicate heating released a gas amount roughly the same as the hot blank, assuring total extraction. Gas purification was achieved with two SAES AP-10-N getter pumps, and helium was measured with a Quadruple Prisma Plus QMG 220. The ^4^He/^3^He ratio of the Q standard (which contains a known amount of ^4^He) mixed with pure ^3^He, which we refer to as the spiked standard, was measured. For the apatite samples, the extracted ^4^He was mixed with the same amount of ^3^He, and the ^4^He/^3^He ratios of the spiked samples were measured. The ^4^He contents (in nano cc) in the samples were calculated using the following equation:

After helium extraction, Pt-wrapped grains were transferred to PFA vials for dissolution following the procedure of ^26^. 25 μL of spike solution (stored in 7 Mol/L HNO_3_) containing 15 ng/mL U and 5 ng/mL Th (^235^U/^238^U = 838 ± 7, ^230^Th/^232^Th = 10.45± 0.05) was added to each vial. To completely dissolve U and Th, the PFA vials were ultrasonically washed for 15 min. After remaining for at least 4 h at room temperature to ensure full dissolution, 325 μL of reagent grade Milli-Q water was added to the vials, and they were diluted to a total volume of 350 μL. All of the spiked samples were measured on a Thermal Fisher X-Series II ICP-MS with suitable parameters choices (Table S2). The same acid as used in the spike solution was utilized as the reagent blank. Durango apatite was used as the standard to verify the analytical procedure. An average age of 32.24 ± 1.01 Ma ^27^ was obtained for the Durango apatite, which is consistent with ages yielded by other laboratories ^28,29^.

**^40^Ar/^39^Ar results**

Raw data for the ^40^Ar/^39^Ar results are shown in Table S3. Characterized by complicated microstructures that serve as domains with different sizes, argon retention properties and closure temperatures ^30-32^, K-feldspars are potentially able to record ^40^Ar/^39^Ar ages of closure temperatures ranging from 350 down to 150 °C as they cool ^33,34^, and thermal histories can consequently be extracted from them ^31,32, 35,36^. Two K-feldspar samples exhibit flat age spectra (Fig. 3), defining good plateaus that account for > 80% of the total ^39^Ar released at high temperature steps. The plateau ages are 232.5±1.4 Ma (15kl03) and 234.6±1.2 Ma (1335-2) , implying that these massifs exhumed rapidly during early Late Triassic, which may be related to the orogeny of the Kunlun Belt.

Conversely, the two age spectra display staircase shapes at the low temperature steps (Fig.3). Although less than 20% of the total released ^39^Ar resides within these low-temperature domains (Fig.3), the behavior ^31, 32,35^ of multi-diffusion domains within K-feldspar suggests that these staircase spectra are the result of slow cooling and, therefore, recorded cooling ages between ~350-150 ^o^C. Their minimum ages of 198.8±2.4 and 90.3±2.1 Ma (Fig.3, Table S1) reflect the final time when the samples passed the ~150 ^o^C isotherm.

In summary, the staircase age spectra at low-temperatures indicate degassing of the smaller and less retentive domains, whereas the flat age spectra at high-temperatures reflect degassing of bigger and stronger retentive domains within K-feldspar. This tallies with a fast, followed by a slow, exhumation of the two samples until a depth where the ambient temperature became close to the closure of the smallest domain (e.g., ~150 ^o^C). The ^40^Ar/^39^Ar thermochronologic data on K-feldspars imply that rocks now at the surface resided at or above ~350 ^o^C during the early Late Triassic and ~150 during late Cretaceous. These results place a maximum bound on total exhumation of ~ 11 km since the middle Triassic and ~5 km since the late Cretaceous, assuming a gradient of 30 ^o^C/km ^13^.

**(U-Th)/He results**

A total of sixty-five apatite grains were dated from fourteen samples along transects 1, 2 and 3, and the raw results are shown in Table S4. Thanks to their ubiquity and moderately high U and Th contents, apatite (U-Th)/He dating is frequently utilized. More importantly, because of the accumulation of helium at temperatures below ~60 ^o^C ^29^ and the removal of helium by diffusion as fast as it is produced by decay at higher temperatures, apatite (U-Th)/He dating results document the latest phase of cooling in the uppermost crust ^37^.

An average age determined from two to six single grain ages analyzed separately from a sample is used as the age of the sample (Table S4). The measurement results show that most samples yielded at least more than two consistent single grain ages consistent for each sample. Age reproducibility is an indispensable demonstration of the quality of an apatite He age ^38^.

For some samples the dates show significant dispersion beyond the analytical precision when an assigned error for the assumptions related to the α-correction is included. Of the fifteen grains that exhibit the over dispersion of the average date in the respective sample they are associated with thirteen are older , and two are younger than the bulk of the remaining dates in those samples. The most possible reason for the older dates is the presence of micro-inclusions with high U and Th concentrations, especially zircon, monazite and uraninite, producing erroneously high He age in some apatites ^39,40^. These inclusions, which are too small to detect by microscopic examination of grains, contribute He to analysis but are not dissolved during the apatite analysis process and hence do not contribute the U and Th contained within them. Inclusions tend to be heterogeneously distributed from grain to grain, causing poor age reproducibility. It has been argued that the presence of inclusions might not be as serious a problem as often thought ^41^, but the presence of unobserved mineral inclusions with high U and Th can easily gain the enrichment factors of 3-4 order of magnitude more U and Th than is present in the apatite, especially for those with relatively low U and Th ^42^. The average U content of all apatites analyzed in this study is 10.6 ppm, and 27.1 ppm Th, with concentrations as low as 0.77 ppm for U and 5.5 ppm for Th, and maxima of 27.1 ppm for U and 79.5 ppm for Th. The effects of the undetected inclusions can be intensified by the effects of α particle implantation from high U and Th minerals contacting to the apatite grain ^39^, and by the effects of U and Th zoning within the apatite ^38,43,44^.

The two much younger dates (1.5±1.3 Ma and 0.2±0.1 Ma from transect 2) are difficult to explain. In some cases, the unaccounted for effects of specific types of U and Th zonation within apatite can lead to younger dates when applying the Ft correction under the assumption of a homogeneous distribution, the effects may exceed a 30% difference in date ^45^. In this case the two dates are significantly younger than that. A possible reason for this is that fractures occur within both grains, which may act as preferential pathway responsible for helium loss ^46^.

Two other reasons for over dispersion of apatite He age are radiation damage ^46-49^ and grain size effect on helium retention ^50^. Both effects are usually considered to be most significant in rocks that experienced relatively slowly cooling through the apatite partial retention zone. The grains of the three samples at the highest elevation reaches of transect 1 show clear correlations between eU and date (Fig. S4a), which could be due to radiation damage effects and are subject to temperatures in the partial retention zone for long period. By contrast, the other samples of transect 1 (Fig. S4b), and all the samples of transect 2 and3 (Fig. S4c), do not exhibit such correlation, suggesting minor effects on the grains of these samples from radiation damage and not suffering slowly cooling. This is consistent with our observation. We see no evidence for the effects of grain size on He retention in the date: the samples where a wide range of grain sizes were analyzed show no correlation between grain size and age (Fig. S5).

To sum up, the dispersion in the dataset is possibly caused by a combination of the effects discussed above, with an emphasis on some radiation damage related effects. The average ages listed in Table S4 exclude the fifteen dates, which are the cause for the over dispersion of the data. The excluding is based on how much older or younger they are compared to the other grains from the same sample. The average dates are standard weighted mean ages, where the analytical uncertainty of each individual analysis was used as the weight.

**Forward modeling**

A one-dimensional thermal model was used for forward modeling of the thermal response to exhumation by using QTQt v.5.3.0 ^51^. A strategy for modeling thermal histories constrained by thermochronologic data from multiple samples in vertical profiles is used, which is preferable in that the final model is easier to interpret and generally has smaller uncertainties than the case in which samples are modeled independently ^51^. Overall, our approach has two aims: 1) to minimize the complexity of the inferred thermal history models, and 2) to model a suite of data from multiple samples jointly by grouping the data. During modeling, the present temperature at the surface is set as 10 ^o^C, the helium-ejection distance is set to 20.0 microns, D_0_ is set to 0.005 m^2^/s, and the activation energy is set to 138000.0 J mol/K.

By using this strategy and parameterization, we first modeled the thermal gradient of the Kunlun Belt under different cooling histories, and a series geothermal gradients from 15 - 50 ^o^C/km were tested (Fig. S6). A best-fit result was obtained for a geothermal gradient of 30 ^o^C/km, which is quite consistent with previous observations ^12,14,52^. Then, various exhumation histories were imposed, assuming a geothermal gradient of 30 °C/km and a constant surface temperature of 10 °C. A range of thermal history solutions for a transect was obtained using a constant temperature offset (30 ^o^C/km) between the top and bottom samples. The detailed modeling procedure can be found in Gallagher et al. ^51^. Samples along a transect were used to calculate model helium ages with helium diffusion kinetics from Flowers et al. ^53^ (radiation damage model RDAMM). We seek a series of preferred model fits that faithfully represent most of the sample ages, i.e., the basic trend of the data.

**Supplementary references**

1. Matte, P. *et al.* Tectonics of Western Tibet, between the Tarim and Indus. Earth and Planetary Science Letters 14, 311–330 (1996).
2. Li, W. et al.. Plaeozoic evolution of the Qimantagh magmatic arcs, Eastern Kunlun Mountains: Constraints from zircon dating of granitoids and modern river sands. Journal of Asian Earth Sciences 77, 183-202 (2013).
3. Dai,J., Wang, C., Hourigan, J. & Santosh M. Multi-stage tectono-magmatic events of the Eastern Kunlun Range, northern Tibet: Insights from U–Pb geochronology and (U–Th)/He thermochronology. Tectonophysics 599, 97–106 (2013).
4. Meng, Q. & Fang, X. Cenozoic tectonic development of the Qaidam Basin in the northeastern Tibetan Plateau in *Investigations into the Tectonics of the Tibetan Plateau* (eds. Burchfi el, B.C., and Wang, E.) 1-24 (Geological Society of America Special Paper 444, 2008).
5. Harris, N.B.W., Ronghua, X., Lewis, C.L., Hawkesworth, C.J. & Yuquan, Z. Isotope Geochemistry of the 1985 Tibet Geotraverse, Lhasa to Golmud. Philosophical Transactions of the Royal Society of London. Series A, Mathematical and Physical Sciences 327, 263–285 (1988).
6. Huang, H. *et al.* Geochemical constraints on the petrogenesis of granitoids in the East Kunlun Orogenic belt, northern Tibetan Plateau: Implications for continental crust growth through syn-collisional felsic magmatism. Chemical Geology 370, 1–18 (2014).
7. Jolivet, M. et al. Mesozoic and Cenozoic tectonics of the northern edge of the Tibetan plateau: fission-track constraints. Tectonophysics 343, 111-134 (2001).
8. Yang, J., Xu, Z., Li, H. & Shi, R. The paleo-Tethyan volcanism and plate tectonic regime in the A'nyemaqen region of East Kunlun , northern Tibet Plateau. Acta Petrologica et Mineralogica 24, 369-380 (2005).
9. Tapponnier, P. *et al.* Propagating extrustion tectonics in Asia: new insights from simple experiments with plasticine. Geology 10, 611–616 (1982).
10. Tapponnier, P., Peltzer, G., Le Dain, A.Y., Armijo, R. & Cobbold, P. Oblique stepwise rise and growth of the Tibet Plateau: Science 294, 1671–1677 (2001).
11. England, P.C. & Houseman, G.A. Finite strain calculations of continental deformation 2. comparison with the India-Asia collision zone. Journal of Geophysical Research 91, 3664-3676 (1986).
12. Wang, C. *et al.* Constraints on the early uplift history of the Tibetan Plateau. Proc. Natl Acad. Sci. USA 105, 4987–4992 (2008).
13. Clark, M.K., Farley, K.A., Zheng, D., Wang, Z. & Duvall, A.R. Early Cenozoic faulting of the northern Tibetan Plateau margin from apatite (U–Th)/He ages. Earth and Planetary Science Letters 296, 78–88 (2010).
14. Chung, S. *et al.* Diachronous uplift of the Tibetan plateau starting 40 Myr ago. Nature 394, 769-773 (1998).
15. Wang, F. *et al.* Relief history and denudation evolution of the northern Tibet margin: Constraints from ^40^Ar/^39^Ar and (U–Th)/He dating and implications for far-field effect of rising plateau. Tectonophysics 675: 196-208 (2016).
16. Rowley, D.B. Age of initiation of collision between India and Asia: a review of stratigraphic data. Earth Planet. Sci. Lett. 145, 1–13 (1996).
17. Ding, L., Kapp, P. & Wan, X.Q. Paleocene– Eocene record of ophiolite obduction and initial India-Asia collision, south central Tibet: Tectonics 24, TC3001 (2005).
18. Wu, F.Y. *et al.* Zircon U–Pb and Hf isotopic constraints on the onset time of India- Asia collision. American Journal of Science 314, 548–579 (2014).
19. Hu, X., Garzanti, E., Moore & T., Raffi, I. Direct stratigraphic dating of India-Asia collision onset at the Selandian (middle Paleocene, 59 ± 1 Ma). Geology 43, 859-862 (2015).
20. Braun, J. van der Beek, P. and Batt, G. Quantitative thermochronology - Numerical method for the interpretation of thermochronological data, 164-171 (Cambridge University , 2006).
21. Chen, L. Research on Late Cenozoic surface denudation and isostatic rebound in eastern Kunlun mountains based on DEM. Master degree dissertation, China University of Geosciences, p.28-42 (2013).
22. Shackleton, R.M., Chang, C. Cenozoic uplift and deformation of the Tibet plateau: the geomorphological evidence in *The geological evolution of the Tibet plateau* (ed. Chang, C.) 327-383 (Scientific Press,1990).
23. [Wang](http://www.sciencedirect.com/science/article/pii/S0009254114004185), F. *et al.* YBCs: A new standard for ^40^Ar/^39^Ar dating. Chemical Geology 388: 87-98 (2014).
24. Wang, F. *et al.* An ^40^Ar/^39^Ar geochronology on a mid-Eocene igneous event on the Barton and Weaver peninsulas: Implication for the dynamic setting of the Antarctic Peninsula. *Geochemistry Geophysics Geosystems*, 10(12): Q12006 (2009).
25. Koppers, A.A.P. ArArCALC-software for ^40^Ar/^39^Ar age calculations. Computers & Geosciences 28, 605-619 (2002).
26. Evans, N.J., Byme, J.P., Keegan, J.T. & Dptter, L.E. Determination of Uranium and Thorium in zircon, apatite, and fluorite: Application to laser (U-Th)/He thermochronology. Journal of Analytical Chemistry, 60 (12), 1159-1165 (2005).
27. Wu, L. *et al.* Cenozoic exhumation history of Sulu terrane: Implications from (U–Th)/He thermochrology. Tectonophysics 672–673, 1–15 (2016).
28. McDowell, F.W., Mdntosh, W.C. & Farley, K.A. A precise 40Ar–39Ar reference age for the Durango apatite (U–Th)/He and fission-track dating standard. Chem. Geol. 214, 249–263 (2005).
29. Reiners, P. W. & Brandon, M. T. Using thermochronology to understand orogenic erosion. Annu. Rev. Earth Planet. Sci. 34, 419-466 (2006).
30. Parsons, I. *et al.* Eight-phase alkali feldspars: low-temperature cryptoperthite, peristerite and multiple replacement reactions in the Klokken intrusion. Contributions to Mineralogy and Petrology 165, 931–960 (2013).
31. Cassata, W.S. & Renne, P.R. Systematic variations of argon diffusion in feldspars and implications for thermochronometry. Geochimica et Cosmochimica Acta 112, 251–287 (2013).
32. Wang, F. *et al.* ^40^Ar/^39^Ar geochronology of the North China and Yangtze Cratons: New constraints on Mesozoic cooling and cratonic destruction under East Asia. Journal of Geophysical Research 119: 3700-3721 (2014).
33. Wartho, J.A. *et al*. Direct measurement of Ar diffusion profiles in a gem-quality Madagascar K-feldspar using the ultra-violet laser ablation microprobe (UVLAMP), Earth and Planetary Science Letters 170, 141-153 (1999).
34. Lovera, O.M., Grove, M. & Harrison, T.M. Systematic analysis of K-feldspar ^40^Ar/^39^Ar step heating results II: Relevance of laboratory argon diffusion properties to nature. Geochimica et Cosmochimica Acta 66, 237–1255 (2002).
35. Lovera, O. M., Richter, F.M. & Harrison, T. M. Diffusion domains determined by ^39^Ar released during step heating, Journal of Geophysical Research 96, 2057-2069 (1991).
36. Lee, J.K.W. Multipath diffusion in geochronology. Contributions to Mineralogy and Petrology 120, 60-82 (1995).
37. Ketcham, R.A., Donelick, D.A. & Carlson, W.D. Variability of apatite Fission-track annealing kinetics: III. Extrapolation to geologic time scales. Am. Mineral. 84, 1235-1255 (1999).
38. Ehlers, T.A. & Farley, K.A. Apatite (U-Th)/He thermochronometry: methods and app;ications to problems in tectonic and surface processes. Earth and Planetary Science Letters 206, 1-4 (2003).
39. Spiegel, C., Kohn, B., Belton, D., Berner, Z. & Gleadow, A. J. W. Apatite (U-Th-Sm)/He thermochronology of rapidly cooled samples: The effect of He implantation. *Earth and* Planetary Science Letters 285, 105-114 (2009).
40. Fitzgerald, P. G., Baldwin, S. L., Webb, L. E. & O'Sullivan, P. B. Interpretation of (U-Th)/He single grain ages from slowly cooled crustal terranes: A case study from the Transantarctic Mountains of southern Victoria Land. Chemical Geology 225, 91-120 (2006).
41. Vermeesch, P. et al. Alpha-emitting mineral inclusions in apatite, their effect on (U-Th)/He ages, and how to reduce it. Geochimica et Cosmochimica Acta 71,1737-1746 (2007).
42. Wang, E. *et al.* Two-phase growth of high topography in eastern Tibet during the Cenozoic. Nature Geoscience 5, 640-645 (2012).
43. Farley, K.A. (U-Th)/He dating: Techniques, calibrations,and applications in *Noble Gas Geochemistry, Reviews in Mineralogy and Geochemistry 47* (eds. Porcelli,P.D., Ballentine,C.J., Wieler R.) 819-843 (2002).
44. Farley, K. A., Shuster, D. L. & Ketcham, R. A. U and Th zonation in apatite observed by laser ablation ICPMS, and implications for the (U-Th)/He system. Geochimica et Cosmochimica Acta 75, 4514-4530 (2011).
45. Hourigan, J. K., Reiners, P. W. & Brandon, M. T. U-Th zonation-dependent alpha-ejection in (U-Th)/He chronometry. Geochimica et Cosmochimica Acta 46, 637-649 (2005).
46. Flowers, R. M., Shuster, D. L., Wernicke, B. P. & Farley, K. A. Radiation damage control on apatite (U-Th)/He dates from the Grand Canyon region, Colorado Plateau. Geology 35, 447-450 (2007).
47. Gautheron, C., Tassan-Got, L., Barbarand, J. & Pagel, M. Effect of alpha-damage annealing on apatite (U-Th)/He thermochronology. Chemical Geology 266, 166-179 (2009).
48. Shuster, D. L. & Farley, K. A. The influence of artifical radiation damage and thermal annealing on helium diffusion kinetics in apatite. Geochimica et Cosmochimica Acta 73, 183-196 (2009).
49. Shuster, D. L., Flowers, R. M. & Farley, K. A. The influence of natural radiation damage on helium diffusion kinetics in apatite. Earth and Planetary Science Letters 249, 148-161 (2006).
50. Reiners, P. W. & Farley, K. A. Influence of crystal size on apatite (U-Th)/He thermochronometry: An example from the Bighorn Mountains, Wyoming. Earth and Planetary Science Letters 188, 413-420 (2001).
51. Gallagher, K., Stephenson, J., Brown, R., Holmes, C. & Fitzgerald, P. Low temperature thermochronology and modeling strategies for multiple samples 1: Vertical profiles. Earth and Planetary Science Letters 237, 193-208 (2005).
52. Qiu, N. Geothermal regime in the Qaidam basin, northern Qinghai-Tibet Plateau. Geol. Mag. 140, 707-719 (2003).
53. Flowers, R.M., Ketcham, R.A., Shuster, D.L. & Farley, K.A. Apatite (U–Th)/He thermochronometry using a radiation damage accumulation and annealing model. Geochemica et Cosmochimica Acta 73, 2347–2365 (2009).

**
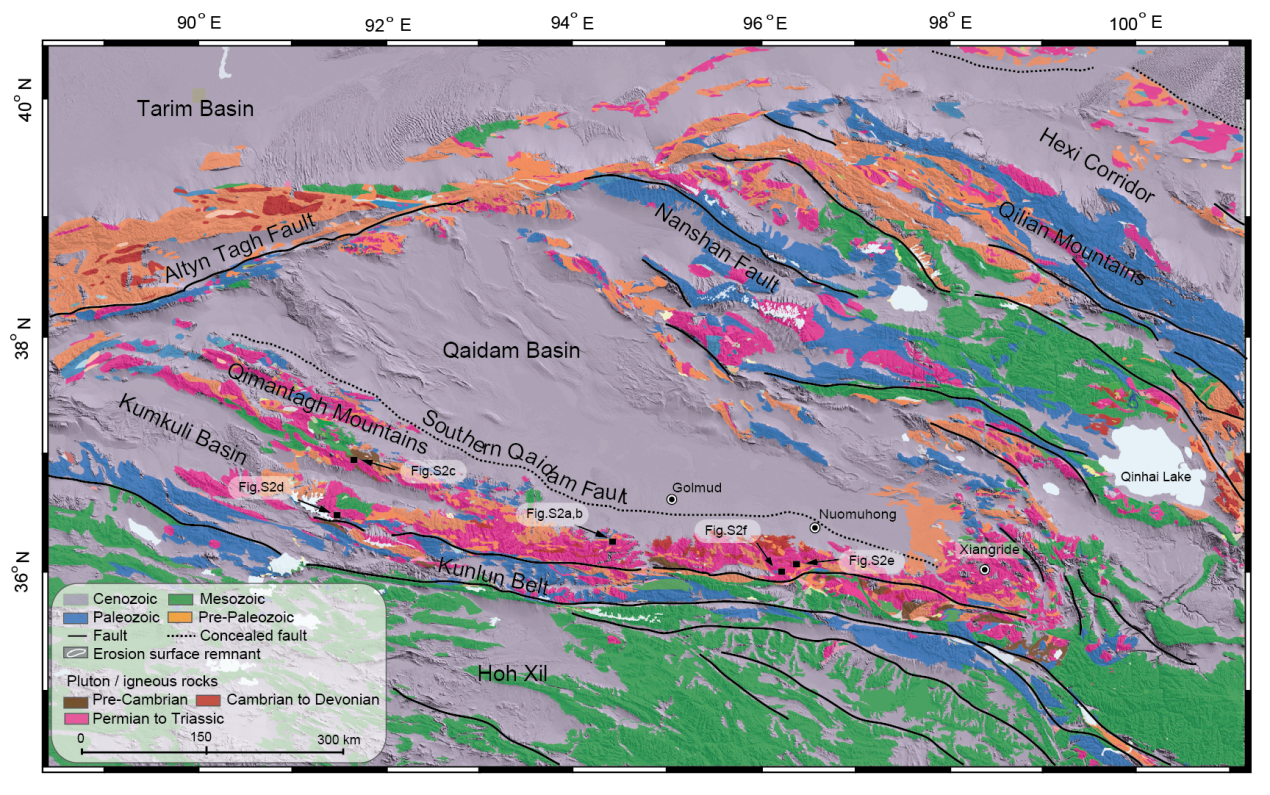
**

**Fig. S1 Geologic map of the study area.** Erosion surface remnants close to transects studied are marked (black squares). Figure was generated using MapInfo Professional [11.0.4], (URL: http://www.pbinsight.com/) and Global Mapper [17.2], (USL: http://www.bluemarblegeo.com). The coordinate system is the World Geodetic System 1984 (WGS84).


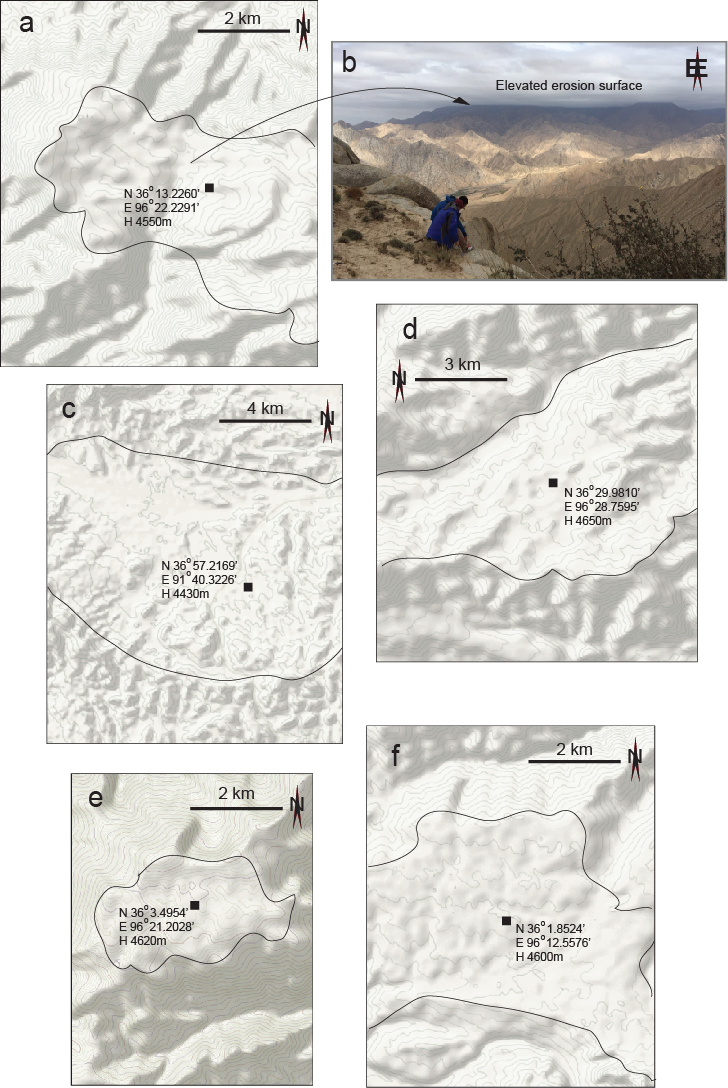


**Fig. S2 Images of erosion surface remnants resolved by using DEM data.** The erosion surface remnants are circled in solid lines. Figures were generated using GoodyGIS [3.25], (USL: http://www.goodygis.com). A position for each surface is marked with latitude, longitude and elevation. a) An erosion surface in middle part of the Kunlun Belt; b) Photograph of erosion surface in (a) shot from the location of transect 1; c) An erosion surface in west part of the Kunlun Belt (Qimantagh); d) An erosion surface in west part of the Kunlun Belt; e) and f) Two erosion surfaces in east part of the Kunlun Belt.


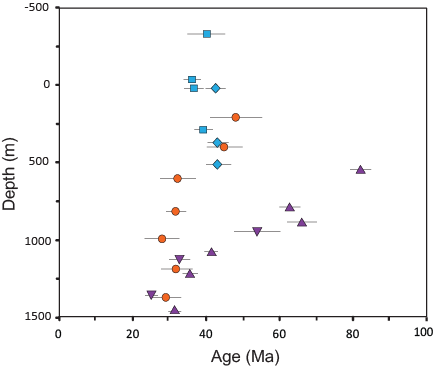


**Fig. S3 Depth-age transects after normalization of elevation relative to the local erosion surface.**


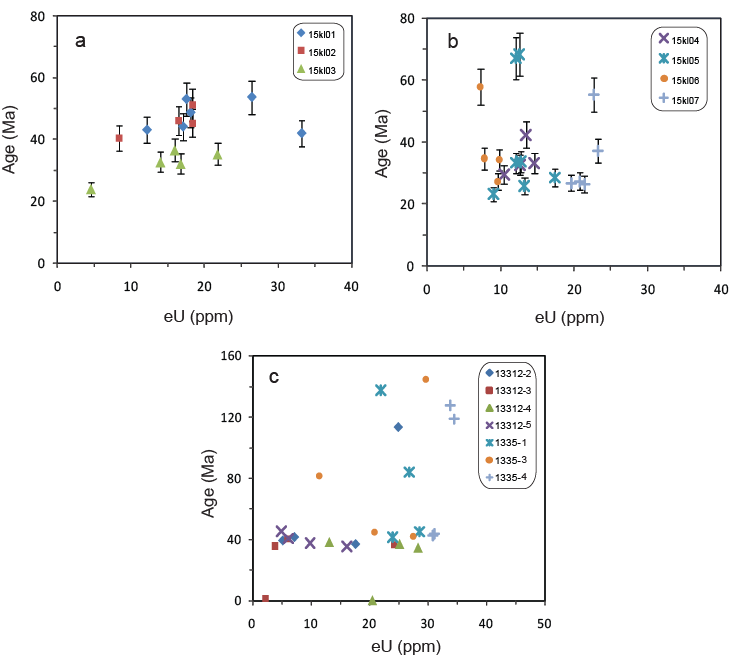


**Fig. S4 Relations between eU and age.** a) Three samples in the highest elevation reaches of transect 1. Positive correlations that could be due to radiation damage effects suggest that the three samples are subject to temperatures in the partial retention zone for long period; b) The rest samples of transect 1, and c) Samples of transects 2 and 3. These samples do not exhibit correlation, suggesting minor effects on the grains from radiation damage and not suffering slowly cooling.


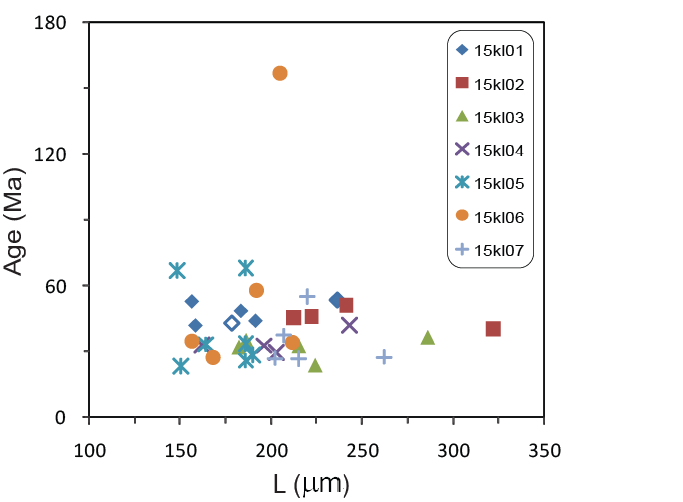


**Fig. S5 Relation between apatite grain length and age.** No correlation between grain size and age is observed, implying that gain size does not affect (U-Th)/He age.


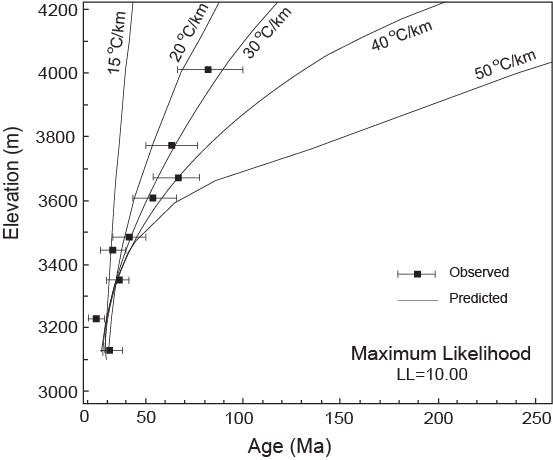


**Fig. S6 Model results of transect 1 compared to age-depth data.** Various geothermal gradients (15, 20, 30, 40, 50 ^o^C/km) were tested in models. Results show that the geothermal gradient of Kunlun belt is most likely 30 ^o^C/km during early Cenozoic.

**Supplementary Table S1**

Summary of sample locations, k-feldspar ^40^Ar/^39^Ar ages and mean apatite (U-Th)/He ages.

| Sample | Longitude | Latitude | Elevation (m) | Depth  (m) | k-feldspar ^40^Ar/^39^Ar Age (Ma) | | | (U-Th)/He Mean Age^a^ (Ma) |
| --- | --- | --- | --- | --- | --- | --- | --- | --- |
|  |  |  |  |  | k-feldspar Plateau Age | k-feldspar smallest domain Age |  | |
| Transect 1 (surface remnant 4550 m) | | | | | | | | |
| 15kl01 | 94.2075 | 36.1849 | 4301 | 249 |  |  | 47.4±5.1 | |
| 15kl02 | 94.2047 | 36.1932 | 4107 | 443 |  |  | 45.7±3.8 | |
| 15kl03 | 94.2061 | 36.1990 | 3902 | 648 | 232.5±1.4 | 90.3±2.1 | 32.1±2.9 | |
| 15kl04 | 94.2097 | 36.2028 | 3687 | 863 |  |  | 31.8±2.2 | |
| 15kl05 | 94.2132 | 36.2027 | 3508 | 1042 |  |  | 28.8±3.1 | |
| 15kl06 | 94.2202 | 36.2079 | 3312 | 1238 |  |  | 32.0±4.5 | |
| 15kl07 | 94.2055 | 36.2383 | 3125 | 1425 |  |  | 29.5±3.3 | |
| Transect 2 (surface remnant 4430 m) | | | | | | | | |
| 13312-2 | 91.6863 | 36.8814 | 4102 | 328 |  |  | 39.3±3.0 | |
| 13312-3 | 91.6740 | 36.9020 | 4370 | 60 |  |  | 37.2±3.5 | |
| 13312-4 | 91.6714 | 36.9102 | 4426 | 4 |  |  | 36.6±3.2 | |
| 13312-5 | 91.6704 | 36.9210 | 4727 | -297 |  |  | 39.8±3.0 | |
| Transect 3 (surface remnant 4650 m) | | | | | | | | |
| 1335-1 | 90.3216 | 36.8650 | 4593 | 57 |  |  | 43.1±4.5 | |
| 1335-2 | 90.3187 | 36.8661 | 4307 | 343 | 234.6±1.2 | 198.8±2.4 | N/A | |
| 1335-3 | 90.3146 | 36.8661 | 4252 | 398 |  |  | 43.2±4.5 | |
| 1335-4 | 90.2596 | 36.9092 | 4112 | 538 |  |  | 43.4±4.4 | |
| Transect 4 ^b^ (surface remnant 4600 m) | | | | | | | | |
| kl22-2 | 95.7200 | 36.2160 | 4010 | 590 |  |  | 82.3±8.6 | |
| kl22-3 | 95.7143 | 36.2190 | 3771 | 829 |  |  | 63.0±6.7 | |
| kl20-2 | 95.7123 | 36.2183 | 3674 | 926 |  |  | 66.5±6.4 | |
| kl21-3 | 95.7049 | 36.2161 | 3481 | 1119 |  |  | 40.8±4.1 | |
| kl21-1 | 95.7054 | 36.2341 | 3351 | 1249 |  |  | 36.0±3.5 | |
| kl18-2 | 95.6902 | 36.2903 | 3130 | 1470 |  |  | 32.0±2.9 | |
| Transect 5 ^b^ (surface remnant 4600 m) | | | | | | | | |
| kl5 | 96.4635 | 36.0575 | 3601 | 999 |  |  | 54.1±5.5 | |
| kl7 | 96.4242 | 36.1046 | 3446 | 1154 |  |  | 33.0±3.1 | |
| kl4-2 | 96.4752 | 36.2186 | 3224 | 1376 |  |  | 24.5±2.0 | |

^a^ Calculated from replicate Ft corrected ^35^ single-grain ages Supplementary Table S4.

^b^ Recalculated from our previous transects in order to make comparison, the raw data can be found in ref. Wang et al. ^15^

**Supplementary Table S2**

ICP-MS parameters

| RF Generator power (W) | 1220 |
| --- | --- |
| Plasma Argon flow (L/min) |  |
| Plasma gas | 13.0 |
| Auxiliary | 0.8 |
| Nebuliser | 1.05 |
| Standard resolution | 125 |
| Data acquisition mode | Peak jump |
| Dwell time (ms) | 10 |
| Sweeps | 100 |
| Number of replicates | 7 |
| Channels | 3 |
| Seperation AMU | 0.02 |
| Analysis time per sample (min) | 1.5 |
| Mass Mnoitored | 230, 232, 234, 235, 238 |

**Supplementary Table S3**

**k-feldspar ^40^Ar/^39^Ar analysis data for samples from transect 2 and 3**

13352 Kfs

| Temp (^o^C) | 36Ar(a) | 37Ar(ca) | 38Ar(cl) | 39Ar(k) | 40Ar(r) | Age (Ma) | ± 2σ   (Ma) |
| --- | --- | --- | --- | --- | --- | --- | --- |
| J = 0.0047500±0.0000119 | | | | | | | |
| 450 | 0.000033 | 0.000520 | 0.000002 | 0.001420 | 0.034736 | 198.83 | 2.40 |
| 450 | 0.000009 | 0.000459 | 0.000000 | 0.001790 | 0.044486 | 201.77 | 1.53 |
| 500 | 0.000017 | 0.000551 | 0.000012 | 0.001964 | 0.053874 | 221.52 | 7.18 |
| 500 | 0.000003 | 0.000490 | 0.000001 | 0.001082 | 0.028796 | 215.21 | 1.91 |
| 550 | 0.000015 | 0.000490 | 0.000002 | 0.003245 | 0.088009 | 219.14 | 1.64 |
| 550 | 0.000009 | 0.000459 | 0.000003 | 0.006311 | 0.172423 | 220.65 | 1.55 |
| 600 | 0.000025 | 0.000490 | 0.000002 | 0.007247 | 0.196481 | 219.06 | 1.43 |
| 600 | 0.000014 | 0.000551 | 0.000005 | 0.010472 | 0.288968 | 222.72 | 1.59 |
| 650 | 0.000045 | 0.000337 | 0.000009 | 0.009462 | 0.267118 | 227.55 | 2.01 |
| 650 | 0.000014 | 0.000282 | 0.000012 | 0.016099 | 0.464103 | 232.06 | 1.51 |
| 700 | 0.000023 | 0.000275 | 0.000014 | 0.016067 | 0.465362 | 233.09 | 1.93 |
| 700 | 0.000038 | 0.000429 | 0.000017 | 0.026335 | 0.763087 | 233.19 | 1.63 |
| 750 | 0.000034 | 0.000367 | 0.000013 | 0.015774 | 0.456728 | 233.02 | 1.54 |
| 750 | 0.000039 | 0.000429 | 0.000010 | 0.016109 | 0.465204 | 232.46 | 1.68 |
| 800 | 0.000033 | 0.000223 | 0.000008 | 0.009931 | 0.284702 | 230.87 | 2.05 |
| 800 | 0.000050 | 0.000306 | 0.000009 | 0.014970 | 0.427603 | 230.08 | 1.58 |
| 850 | 0.000050 | 0.000266 | 0.000010 | 0.011427 | 0.326220 | 229.95 | 1.52 |
| 850 | 0.000078 | 0.000337 | 0.000014 | 0.017723 | 0.504912 | 229.50 | 1.59 |
| 875 | 0.000038 | 0.000297 | 0.000006 | 0.009090 | 0.257981 | 228.69 | 1.67 |
| 875 | 0.000076 | 0.000251 | 0.000015 | 0.018933 | 0.542206 | 230.63 | 1.77 |
| 900 | 0.000024 | 0.000306 | 0.000005 | 0.006946 | 0.197668 | 229.27 | 2.17 |
| 925 | 0.000032 | 0.000367 | 0.000006 | 0.009878 | 0.282973 | 230.71 | 1.92 |
| 950 | 0.000039 | 0.000294 | 0.000006 | 0.012515 | 0.357664 | 230.19 | 1.72 |
| 975 | 0.000044 | 0.000398 | 0.000010 | 0.013385 | 0.383051 | 230.48 | 1.63 |
| 1000 | 0.000043 | 0.000429 | 0.000009 | 0.012611 | 0.362601 | 231.50 | 1.65 |
| 1025 | 0.000050 | 0.000337 | 0.000014 | 0.017187 | 0.495220 | 231.96 | 1.61 |
| 1050 | 0.000046 | 0.000223 | 0.000011 | 0.014732 | 0.426204 | 232.85 | 1.63 |
| 1075 | 0.000052 | 0.000306 | 0.000015 | 0.014918 | 0.433244 | 233.69 | 1.75 |
| 1100 | 0.000061 | 0.000429 | 0.000014 | 0.019305 | 0.558663 | 232.91 | 1.56 |
| 1100 | 0.000096 | 0.000398 | 0.000023 | 0.047132 | 1.374514 | 234.60 | 1.47 |
| 1100 | 0.000076 | 0.000196 | 0.000024 | 0.037867 | 1.104751 | 234.68 | 1.61 |
| 1100 | 0.000086 | 0.000367 | 0.000021 | 0.040124 | 1.168005 | 234.20 | 1.49 |
| 1100 | 0.000078 | 0.000337 | 0.000016 | 0.029663 | 0.864962 | 234.57 | 1.54 |
| 1100 | 0.000122 | 0.000254 | 0.000019 | 0.038050 | 1.107615 | 234.19 | 1.56 |
| 1200 | 0.000053 | 0.000275 | 0.000022 | 0.026786 | 0.779842 | 234.23 | 1.83 |
| 1225 | 0.000057 | 0.000367 | 0.000022 | 0.035190 | 1.026539 | 234.66 | 1.45 |
| 1250 | 0.000054 | 0.000398 | 0.000021 | 0.032771 | 0.957802 | 235.08 | 1.43 |
| 1300 | 0.000044 | 0.000337 | 0.000011 | 0.021256 | 0.620663 | 234.88 | 1.54 |

15kl03 Kfs

| Temp (^o^C) | 36Ar(a) | 37Ar(ca) | 38Ar(cl) | 39Ar(k) | 40Ar(r) | Age (Ma) | ± 2σ   (Ma) |
| --- | --- | --- | --- | --- | --- | --- | --- |
| J = 0.0041960±0.0000105 | | | | | | | |
| 450 | 0.000369 | 0.000025 | 0.000000 | 0.000059 | 0.009858 | 1024.09 | 278.86 |
| 450 | 0.000043 | 0.000024 | 0.000000 | 0.000130 | 0.003590 | 214.18 | 23.13 |
| 500 | 0.000018 | 0.000014 | 0.000000 | 0.000246 | 0.003314 | 107.36 | 10.11 |
| 500 | 0.000008 | 0.000067 | 0.000002 | 0.000713 | 0.008042 | 90.25 | 2.06 |
| 550 | 0.000034 | 0.000161 | 0.000003 | 0.001667 | 0.021939 | 104.90 | 2.59 |
| 550 | 0.000078 | 0.000358 | 0.000003 | 0.004554 | 0.062891 | 109.92 | 1.33 |
| 600 | 0.000091 | 0.000329 | 0.000003 | 0.007394 | 0.111179 | 119.36 | 1.16 |
| 600 | 0.000034 | 0.000294 | 0.000004 | 0.014502 | 0.235556 | 128.61 | 0.98 |
| 650 | 0.000013 | 0.000209 | 0.000001 | 0.014376 | 0.258760 | 141.99 | 1.08 |
| 650 | 0.000012 | 0.000209 | 0.000002 | 0.018030 | 0.373733 | 162.57 | 1.32 |
| 700 | 0.000013 | 0.000175 | 0.000001 | 0.012761 | 0.301416 | 184.13 | 1.30 |
| 700 | 0.000013 | 0.000175 | 0.000001 | 0.016181 | 0.414865 | 199.03 | 2.96 |
| 750 | 0.000015 | 0.000163 | 0.000002 | 0.011595 | 0.320507 | 213.69 | 1.93 |
| 750 | 0.000015 | 0.000140 | 0.000003 | 0.016206 | 0.454836 | 216.79 | 1.62 |
| 800 | 0.000015 | 0.000117 | 0.000000 | 0.012869 | 0.368360 | 220.84 | 1.56 |
| 800 | 0.000018 | 0.000107 | 0.000002 | 0.020218 | 0.586346 | 223.58 | 1.48 |
| 850 | 0.000027 | 0.000190 | 0.00002 | 0.024426 | 0.718777 | 226.66 | 1.47 |
| 850 | 0.000042 | 0.000181 | 0.00000 | 0.044027 | 1.297149 | 226.92 | 1.42 |
| 875 | 0.000028 | 0.000154 | 0.000003 | 0.029737 | 0.887351 | 229.65 | 1.39 |
| 875 | 0.000058 | 0.000275 | 0.000001 | 0.075075 | 2.244782 | 230.09 | 1.37 |
| 900 | 0.000016 | 0.000169 | 0.000002 | 0.029381 | 0.882544 | 231.08 | 1.43 |
| 925 | 0.000018 | 0.000242 | 0.000003 | 0.038348 | 1.149850 | 230.70 | 1.39 |
| 950 | 0.000016 | 0.000182 | 0.000004 | 0.049464 | 1.481127 | 230.40 | 1.41 |
| 975 | 0.000017 | 0.000130 | 0.000006 | 0.053316 | 1.601077 | 231.03 | 1.41 |
| 1000 | 0.000011 | 0.000091 | 0.000001 | 0.048502 | 1.464336 | 232.19 | 1.61 |
| 1025 | 0.000009 | 0.000104 | 0.000004 | 0.036088 | 1.087715 | 231.82 | 1.41 |
| 1050 | 0.000007 | 0.000017 | 0.000001 | 0.029553 | 0.891132 | 231.92 | 1.51 |
| 1075 | 0.000008 | 0.000046 | 0.000002 | 0.029114 | 0.884534 | 233.56 | 1.42 |
| 1100 | 0.000008 | 0.000061 | 0.000001 | 0.028929 | 0.882572 | 234.47 | 1.49 |
| 1100 | 0.000011 | 0.000017 | 0.000000 | 0.036522 | 1.115533 | 234.74 | 1.42 |
| 1100 | 0.000011 | 0.000065 | 0.000001 | 0.029490 | 0.901552 | 234.93 | 1.59 |
| 1100 | 0.000021 | 0.000059 | 0.000002 | 0.053952 | 1.640562 | 233.75 | 1.41 |
| 1100 | 0.000018 | 0.000135 | 0.000003 | 0.057444 | 1.748448 | 233.97 | 1.40 |
| 1100 | 0.000029 | 0.000049 | 0.000004 | 0.031984 | 0.976786 | 234.70 | 1.44 |
| 1200 | 0.000005 | 0.000010 | 0.000006 | 0.003826 | 0.117006 | 235.03 | 3.86 |
| 1225 | 0.000004 | 0.000004 | 0.000011 | 0.003233 | 0.099743 | 236.97 | 2.18 |
| 1250 | 0.000004 | 0.000000 | 0.000008 | 0.001632 | 0.049169 | 231.71 | 2.81 |
| 1300 | 0.000006 | 0.000003 | 0.000000 | 0.000287 | 0.023111 | 563.81 | 37.38 |

Notes: ^36^Ar(a),^37^Ar(ca),^38^Ar(cl), ^39^Ar(k),^40^Ar(r) are measured in volts; the ages are calculated using decay constant of λ=5.543x10^-10^yr^-1^ against the standard YBCs (29.286 ± 0.045 Ma) ^23^.

**Supplementary Table S4**

(U-Th)/He analysis data

| **Sample** | **^238^U (mole)** | **±2σ** | **^232^Th (mole)** | **±2σ** | **^4^He (mole)** | **±2σ** | **Age (Ma)** | **±2σ** | **Ft** | **Cor. Age (Ma)** | **±2σ^a^** | **W ^b^**  **(μm)** | **L ^b^**  **(μm)** | **Use^c^** |
| --- | --- | --- | --- | --- | --- | --- | --- | --- | --- | --- | --- | --- | --- | --- |
| **Transect 1** |  |  |  |  |  |  |  |  |  |  |  |  |  |  |
| 15KL01-G1 | 8.6559E-14 | 1.2216E-15 | 2.2058E-13 | 4.7464E-15 | 6.1938E-15 | 9.2249E-17 | 35.02 | 0.67 | 0.66 | 53.06 | 2.84 | 75 | 156 | y |
| 15KL01-G2 | 2.5976E-13 | 4.2542E-15 | 6.1680E-13 | 6.3206E-15 | 1.9669E-14 | 2.8246E-16 | 38.02 | 0.69 | 0.708 | 53.70 | 2.86 | 86 | 236 | y |
| 15KL01-G3 | 1.3729E-13 | 1.8635E-15 | 3.5764E-13 | 4.5262E-15 | 8.7950E-15 | 1.3094E-16 | 31.11 | 0.55 | 0.705 | 44.13 | 2.34 | 88 | 191 | y |
| 15KL01-G4 | 1.4293E-13 | 2.0132E-15 | 3.5164E-13 | 4.7973E-15 | 7.7113E-15 | 1.1372E-16 | 26.77 | 0.48 | 0.638 | 41.96 | 0.60 | 69 | 158 | y |
| 15KL01-G5 | 9.8221E-14 | 1.6058E-15 | 2.5898E-13 | 2.2265E-15 | 6.5231E-15 | 1.0344E-16 | 32.10 | 0.61 | 0.659 | 48.71 | 2.61 | 74 | 183 | y |
| 15KL01-G6 | 7.8855E-14 | 1.1778E-15 | 2.0956E-13 | 1.7210E-15 | 4.8169E-15 | 7.6180E-17 | 29.44 | 0.55 | 0.684 | 43.04 | 2.30 | 82 | 178 | y |
|  |  |  |  |  |  |  |  |  |  |  |  |  |  |  |
| 15KL02-G1 | 1.7921E-13 | 2.6622E-15 | 4.1226E-13 | 4.3375E-15 | 1.0925E-14 | 1.5673E-16 | 30.96 | 0.55 | 0.766 | 40.42 | 2.14 | 108 | 322 | y |
| 15KL02-G2 | 1.5131E-13 | 2.0937E-15 | 3.7938E-13 | 4.1860E-15 | 9.7214E-15 | 1.3986E-16 | 31.64 | 0.55 | 0.698 | 45.33 | 2.40 | 83 | 212 | y |
| 15KL02-G3 | 2.0529E-13 | 2.8373E-15 | 4.0691E-13 | 3.8073E-15 | 1.3011E-14 | 1.8675E-16 | 33.81 | 0.59 | 0.734 | 46.06 | 2.44 | 97 | 222 | y |
| 15KL02-G4 | 1.7186E-13 | 2.4155E-15 | 4.3010E-13 | 4.9901E-15 | 1.2531E-14 | 1.7697E-16 | 35.93 | 0.62 | 0.702 | 51.18 | 2.71 | 84 | 241 | y |
|  |  |  |  |  |  |  |  |  |  |  |  |  |  |  |
| 15KL03-G1 | 1.2848E-13 | 1.8722E-15 | 2.9654E-13 | 5.3955E-15 | 5.6799E-15 | 7.2178E-17 | 22.44 | 0.38 | 0.698 | 32.15 | 1.70 | 86 | 182 | y |
| 15KL03-G2 | 3.5919E-14 | 6.8727E-16 | 7.8577E-14 | 1.6841E-15 | 1.1314E-15 | 1.5019E-17 | 16.29 | 0.32 | 0.682 | 23.89 | 1.28 | 78 | 224 | y |
| 15KL03-G3 | 2.6569E-13 | 3.7970E-15 | 5.0305E-13 | 5.5912E-15 | 1.3383E-14 | 1.9717E-16 | 27.27 | 0.49 | 0.745 | 36.60 | 1.94 | 98 | 286 | y |
| 15KL03-G5 | 1.7160E-13 | 2.3894E-15 | 5.5238E-13 | 4.6012E-15 | 9.6377E-15 | 1.5175E-16 | 25.06 | 0.45 | 0.711 | 35.25 | 1.87 | 91 | 186 | y |
| 15KL03-G6 | 1.4417E-13 | 2.1392E-15 | 2.9670E-13 | 2.5239E-15 | 6.4064E-15 | 7.8044E-17 | 23.44 | 0.38 | 0.717 | 32.69 | 1.72 | 90 | 215 | y |
|  |  |  |  |  |  |  |  |  |  |  |  |  |  |  |
| 15KL04-G1 | 1.6107E-13 | 2.5361E-15 | 3.5313E-13 | 4.6483E-15 | 9.5808E-15 | 1.3713E-16 | 30.71 | 0.56 | 0.725 | 42.36 | 0.55 | 92 | 243 | x |
| 15KL04-G2 | 8.2214E-14 | 1.2898E-15 | 2.1710E-13 | 2.3148E-15 | 3.8042E-15 | 5.4072E-17 | 22.37 | 0.40 | 0.674 | 33.19 | 1.76 | 80 | 162 | y |
| 15KL04-G3 | 6.7305E-14 | 9.6855E-16 | 1.4946E-13 | 2.0386E-15 | 2.5654E-15 | 4.4507E-17 | 19.61 | 0.40 | 0.665 | 29.49 | 1.59 | 74 | 203 | y |
| 15KL04-G4 | 1.0560E-13 | 1.7583E-15 | 2.3561E-13 | 2.5876E-15 | 4.7137E-15 | 8.0986E-17 | 22.92 | 0.47 | 0.701 | 32.70 | 1.77 | 86 | 196 | y |
|  |  |  |  |  |  |  |  |  |  |  |  |  |  |  |
| 15KL05-G1 | 8.8132E-14 | 1.3914E-15 | 1.9954E-13 | 5.4116E-15 | 8.0824E-15 | 9.8668E-17 | 46.78 | 0.86 | 0.685 | 68.29 | 0.60 | 81 | 186 | x |
| 15KL05-G2 | 3.5880E-14 | 5.6575E-16 | 9.7467E-14 | 1.8285E-15 | 1.0997E-15 | 1.6776E-17 | 14.66 | 0.29 | 0.634 | 23.12 | 1.24 | 70 | 150 | y |
| 15KL05-G3 | 7.2963E-14 | 1.2201E-15 | 1.5408E-13 | 1.5988E-15 | 2.3388E-15 | 3.4732E-17 | 16.78 | 0.32 | 0.65 | 25.82 | 1.38 | 71 | 186 | y |
| 15KL05-G4 | 5.5684E-14 | 9.6624E-16 | 1.1592E-13 | 1.3765E-15 | 4.3414E-15 | 6.4355E-17 | 40.92 | 0.79 | 0.611 | 66.97 | 3.59 | 64 | 148 | x |
| 15KL05-G5 | 1.7012E-13 | 2.2693E-15 | 3.5225E-13 | 3.8328E-15 | 8.1689E-15 | 1.2825E-16 | 25.28 | 0.47 | 0.752 | 33.62 | 1.79 | 109 | 186 | y |
| 15KL05-G6 | 1.2633E-13 | 4.0590E-15 | 1.7385E-13 | 1.5661E-15 | 4.0988E-15 | 6.5566E-17 | 19.17 | 0.56 | 0.674 | 28.44 | 1.65 | 76 | 190 | y |
| 15KL05-G7 | 8.4365E-14 | 1.2040E-15 | 1.8983E-13 | 2.4818E-15 | 3.7963E-15 | 5.3352E-17 | 23.04 | 0.40 | 0.694 | 33.20 | 1.76 | 85 | 164 | y |
|  |  |  |  |  |  |  |  |  |  |  |  |  |  |  |
| 15KL06-G3 | 5.8451E-14 | 9.0270E-16 | 1.3221E-13 | 2.4144E-15 | 4.6037E-15 | 7.6749E-17 | 40.21 | 0.82 | 0.696 | 57.77 | 3.12 | 84 | 192 | x |
| 15KL06-G4 | 9.220E-14 | 1.551E-15 | 1.930E-13 | 2.001E-15 | 9.330E-15 | 3.103E-16 | 52.97 | 2.14 | 0.676 | 78.35 | 8.48 | 77 | 205 | x |
| 15KL06-G5 | 7.3239E-14 | 1.2913E-15 | 1.8091E-13 | 1.9470E-15 | 2.8283E-15 | 3.9959E-17 | 19.14 | 0.35 | 0.704 | 27.19 | 1.45 | 89 | 168 | y |
| 15KL06-G6 | 4.1129E-14 | 6.5415E-16 | 8.8778E-14 | 1.0641E-15 | 1.8144E-15 | 2.8496E-17 | 22.91 | 0.44 | 0.661 | 34.66 | 1.86 | 75 | 156 | y |
| 15KL06-G7 | 9.1457E-14 | 1.2716E-15 | 2.1520E-13 | 2.0618E-15 | 4.3935E-15 | 6.7955E-17 | 24.22 | 0.44 | 0.709 | 34.16 | 1.82 | 88 | 212 | y |
|  |  |  |  |  |  |  |  |  |  |  |  |  |  |  |
| 15KL07-G2 | 1.4579E-13 | 2.2377E-15 | 2.7672E-13 | 3.6289E-15 | 4.9239E-15 | 7.8131E-17 | 18.28 | 0.36 | 0.681 | 26.84 | 0.60 | 78 | 202 | y |
| 15KL07-G3 | 2.4574E-13 | 3.2743E-15 | 4.7352E-13 | 4.3666E-15 | 1.2227E-14 | 1.9322E-16 | 26.79 | 0.50 | 0.721 | 37.16 | 0.60 | 92 | 207 | y |
| 15KL07-G4 | 2.0057E-13 | 3.3012E-15 | 3.8836E-13 | 4.7521E-15 | 6.9447E-15 | 1.0081E-16 | 18.63 | 0.35 | 0.704 | 26.46 | 0.60 | 85 | 215 | y |
| 15KL07-G6 | 1.8689E-13 | 2.4674E-15 | 3.3645E-13 | 2.6379E-15 | 1.2864E-14 | 2.2138E-16 | 37.80 | 0.74 | 0.684 | 55.26 | 2.97 | 77 | 220 | x |
| 15KL07-G7 | 5.2781E-13 | 7.0438E-15 | 8.6908E-13 | 7.2391E-15 | 2.0176E-14 | 3.0443E-16 | 21.56 | 0.39 | 0.788 | 27.36 | 1.45 | 123 | 262 | y |

| **Transect 2** |  |  |  |  |  |  |  |  |  |  |  |  |  |  |
| --- | --- | --- | --- | --- | --- | --- | --- | --- | --- | --- | --- | --- | --- | --- |
| 13312-2-G1 | 1.3269E-13 | 3.1229E-15 | 4.7878E-13 | 4.7505E-15 | 2.4439E-14 | 8.7334E-17 | 77.90 | 1.09 | 0.686 | 113.6 | 1.7 | 79 | 175 | x |
| 13312-2-G2 | 5.1819E-14 | 1.8066E-15 | 4.1145E-13 | 3.2841E-15 | 5.6018E-15 | 3.2348E-17 | 29.67 | 1.16 | 0.801 | 37.0 | 1.7 | 77 | 157 | y |
| 13312-2-G3 | 7.7273E-14 | 1.8342E-15 | 1.3924E-13 | 2.0004E-15 | 4.6077E-15 | 2.9260E-17 | 32.75 | 0.6 | 0.787 | 41.6 | 1.1 | 90 | 221 | y |
| 13312-2-G4 | 3.6826E-14 | 6.7848E-16 | 7.9218E-14 | 1.7088E-15 | 1.6807E-15 | 2.0913E-17 | 23.73 | 0.45 | 0.603 | 39.4 | 0.7 | 73 | 230 | y |
|  |  |  |  |  |  |  |  |  |  |  |  |  |  |  |
| 13312-3-G1 | 1.2244E-13 | 1.6675E-15 | 1.3240E-13 | 1.5721E-15 | 5.4192E-15 | 3.4525E-17 | 27.56 | 0.46 | 0.686 | 40.2 | 0.7 | 114 | 231 | y |
| 13312-3-G2 | 5.6835E-15 | 3.4041E-16 | 4.4419E-14 | 5.4061E-16 | 1.9636E-17 | 1.6623E-17 | 0.96 | 0.93 | 0.624 | 1.5 | 1.3 | 69 | 173 | x |
| 13312-3-G3 | 5.2693E-14 | 6.1482E-16 | 1.1973E-13 | 1.5226E-15 | 2.4589E-15 | 2.3801E-17 | 23.81 | 0.31 | 0.667 | 35.7 | 0.4 | 104 | 226 | y |
| 13312-3-G4 | 1.5792E-13 | 1.2475E-15 | 9.6087E-14 | 2.1898E-15 | 5.3023E-15 | 2.9040E-17 | 22.92 | 0.21 | 0.632 | 36.3 | 0.3 | 67 | 186 | y |
|  |  |  |  |  |  |  |  |  |  |  |  |  |  |  |
| 13312-4-G1 | 8.4645E-14 | 6.0502E-16 | 3.9197E-13 | 4.3756E-15 | 2.6744E-17 | 1.6416E-17 | 0.12 | 0.07 | 0.63 | 0.2 | 0.1 | 76 | 170 | x |
| 13312-4-G2 | 2.3432E-13 | 2.6084E-15 | 9.4619E-13 | 1.0252E-14 | 1.3708E-14 | 5.8124E-17 | 23.55 | 0.21 | 0.681 | 34.6 | 0.3 | 96 | 199 | y |
| 13312-4-G3 | 6.0131E-14 | 1.6963E-15 | 4.1568E-13 | 2.0633E-15 | 4.6255E-15 | 3.0692E-17 | 23.05 | 0.2 | 0.604 | 38.2 | 0.3 | 88 | 150 | y |
| 13312-4-G4 | 2.4772E-13 | 1.6640E-15 | 1.0425E-12 | 5.6109E-15 | 1.5173E-14 | 5.9603E-17 | 24.17 | 0.14 | 0.653 | 37.0 | 0.2 | 100 | 223 | y |
|  |  |  |  |  |  |  |  |  |  |  |  |  |  |  |
| 13312-5-G1 | 2.4602E-14 | 7.2868E-16 | 1.5313E-13 | 2.3637E-15 | 1.5030E-15 | 1.9561E-17 | 19.50 | 0.24 | 0.479 | 40.7 | 0.4 | 86 | 156 | y |
| 13312-5-G2 | 4.8023E-14 | 1.0185E-15 | 2.7179E-13 | 1.9061E-15 | 2.8280E-15 | 2.4975E-17 | 19.86 | 0.27 | 0.525 | 37.8 | 0.4 | 95 | 142 | y |
| 13312-5-G3 | 2.3345E-14 | 4.1799E-16 | 1.4143E-13 | 2.4460E-15 | 1.6932E-15 | 1.9473E-17 | 23.52 | 0.31 | 0.518 | 45.4 | 0.4 | 102 | 152 | y |
| 13312-5-G4 | 2.7349E-14 | 1.0298E-15 | 1.9666E-13 | 3.3088E-15 | 1.7609E-15 | 2.1266E-17 | 18.83 | 0.36 | 0.532 | 35.4 | 0.6 | 63 | 127 | y |
|  |  |  |  |  |  |  |  |  |  |  |  |  |  |  |
| **Transect 3** |  |  |  |  |  |  |  |  |  |  |  |  |  |  |
| 1335-1-G1 | 7.9908E-14 | 1.2334E-15 | 2.1243E-13 | 2.5912E-15 | 5.3974E-15 | 3.1310E-17 | 32.55 | 0.39 | 0.789 | 41.3 | 0.6 | 65 | 143 | y |
| 1335-1-G2 | 2.4914E-13 | 1.5976E-15 | 6.1393E-13 | 7.7252E-15 | 3.2418E-14 | 1.0702E-16 | 64.35 | 0.45 | 0.767 | 83.9 | 0.7 | 94 | 190 | x |
| 1335-1-G3 | 2.41E-13 | 4.13E-15 | 7.74E-13 | 5.02E-15 | 5.32E-14 | 4.22E-16 | 98.14 | 2.63 | 0.713 | 137.64 | 3.8 | 96 | 211 | x |
| 1335-1-G4 | 5.9922E-14 | 1.8113E-15 | 3.2730E-13 | 3.2569E-15 | 5.9589E-15 | 3.6498E-17 | 34.19 | 0.67 | 0.761 | 44.9 | 1.1 | 70 | 111 | y |
|  |  |  |  |  |  |  |  |  |  |  |  |  |  |  |
| 1335-3-G1 | 3.0119E-13 | 1.0973E-14 | 5.9036E-13 | 5.2213E-15 | 6.2780E-14 | 2.0696E-16 | 110.96 | 2.84 | 0.767 | 144.7 | 3.9 | 90 | 210 | x |
| 1335-3-G2 | 9.3271E-14 | 6.6500E-16 | 3.7070E-13 | 3.1246E-15 | 1.1941E-14 | 4.6679E-17 | 51.85 | 0.35 | 0.636 | 81.5 | 0.5 | 97 | 192 | x |
| 1335-3-G3 | 1.6138E-13 | 2.6949E-15 | 7.3157E-13 | 1.0579E-14 | 1.5060E-14 | 5.6091E-17 | 35.43 | 0.43 | 0.791 | 44.8 | 0.6 | 94 | 206 | y |
| 1335-3-G4 | 7.1976E-14 | 9.4114E-16 | 2.5057E-13 | 4.0498E-15 | 5.4418E-15 | 3.5991E-17 | 32.58 | 0.49 | 0.772 | 42.2 | 0.8 | 62 | 140 | y |
|  |  |  |  |  |  |  |  |  |  |  |  |  |  |  |
| 1335-4-G1 | 3.4565E-13 | 3.3397E-15 | 5.0937E-13 | 5.2059E-15 | 1.9223E-14 | 1.4433E-16 | 32.27 | 0.7 | 0.754 | 42.8 | 1.0 | 94 | 196 | y |
| 1335-4-G2 | 2.6326E-13 | 2.3568E-15 | 1.4423E-12 | 8.4703E-15 | 6.9670E-14 | 2.3750E-16 | 90.55 | 0.56 | 0.708 | 127.9 | 0.8 | 98 | 193 | x |
| 1335-4-G3 | 2.72E-13 | 3.12E-15 | 3.25E-13 | 2.90E-15 | 3.81E-14 | 1.40E-15 | 85.03 | 8.47 | 0.716 | 118.8 | 12 | 86 | 157 | x |
| 1335-4-G4 | 5.5997E-13 | 6.0428E-15 | 1.3608E-12 | 8.6083E-15 | 3.4329E-14 | 2.8382E-16 | 30.53 | 0.63 | 0.694 | 44.0 | 0.9 | 108 | 268 | y |

^a^ Anaytical error only.

^b^ Dimensions for apatite prism: W, width; L, length.

^c^ y means those used for calculation of mean age, while x means not.
